# Development of Microneedles for Antimicrobial Drug Delivery: A Comprehensive Review on Applications in Wound Infection Management

**DOI:** 10.1002/smsc.202400158

**Published:** 2024-07-21

**Authors:** Hanif Haidari, Richard Bright, Yunlong Yu, Krasimir Vasilev, Zlatko Kopecki

**Affiliations:** ^1^ Future Industries Institute University of South Australia Mawson Lakes SA 5095 Australia; ^2^ College of Medicine and Public Health Flinders University Bedford Park SA 5042 Australia; ^3^ Institute of Burn Research, Southwest Hospital Third Military Medical University (Army Medical University) Chongqing 400038 P. R. China

**Keywords:** antimicrobial microneedles, bacterial biofilms, smart microneedles, targeted deliveries, wound dressings, wound healings, wound infections

## Abstract

Microneedles (MNs) have emerged as a promising transdermal antimicrobial delivery system, providing precise and localized drug delivery while complemented with noninvasiveness and patient compliance. Currently, the topical application of antimicrobials restricts the delivery of drugs to the critical areas of the wound bed, largely due to barriers posed by the necrotic tissue, scab formation, and bacterial biofilms, which severely diminish the bioavailability of the therapeutics. MNs have enabled efficient and targeted delivery to overcome many chronic wound challenges. Over the past decade, significant progress has been made to develop MNs with unique properties tailored for the delivery of vaccines, anticancer, and antimicrobials. As ongoing research continues to refine MN design, material properties, and drug formulations, the potential for revolutionizing antimicrobial drug delivery for efficacy, patient experience, and therapeutic outcomes remains at the forefront of scientific research. In this review, insights are provided into the latest progress, current developments, and the diverse applications of MNs for antimicrobial drug delivery. Herein, the translational potential of MNs is highlighted and a perspective on the current challenges associated with clinical translation is provided. Furthermore, this review aids in identifying research gaps while empowering and contributing to the future implementation of cutting‐edge delivery systems to effectively tackle antimicrobial resistance.

## Graphical abstract

Infected wounds present a significant challenge in healthcare, leading to prolonged inflammation, heightened pain, impaired healing, and increased mortality. In this review, a critical overview of the design strategies for microneedle antimicrobial drug delivery in context of wound management is provided. In addition, current challenges associated with clinical translation are discussed and research gaps in current knowledge are identified.

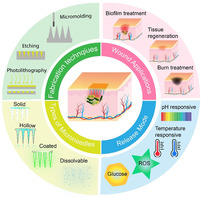

## 1 Introduction

The challenges associated with clinical infection of chronic wounds have become critical issues in contemporary healthcare, including diabetic wounds, burn injuries, pressure ulcers, etc.^[^ ^1^ ^]^ In particular, impaired healing caused by bacterial infections not only compromises the patient's quality of life but also imposes a substantial medical and financial burden on the healthcare system.^[^ ^2^ ^]^ Additionally, the prevalence of chronic wounds is on the rise, driven by the increasing occurrences of obesity, diabetes, and antimicrobial resistance (AMR), alongside the aging populations and extended life expectancy.^[^ ^3^, ^4^ ^]^ Moreover, the discovery and approvals of new antibiotics have severely slowed in the past two decades mostly due to insufficient or unsatisfactory return on investment.^[^ ^5^ ^]^ This has compelled industries and researchers to seek alternative approaches to the management of bacterial wound infection. Wound healing is a complex process involving interconnected processes including hemostasis, inflammation, proliferation, and remodeling.^[^ ^6^ ^]^ The process becomes disrupted when the wound fails to repair in an orderly and timely manner, leading to an abnormal wound. The major impediment in the wound healing cascade is the bacterial infection that disrupts healing and prolongs inflammation. The impaired physiological processes associated with chronic wounds create an environment conducive to bacterial biofilm formation which can significantly impact wound repair.^[^ ^7^ ^]^

In chronic wounds, bacteria adopt a biofilm structure, tightly adhering to the wound bed surface and binding to extracellular polymeric substances (EPS), resulting in a thick structure of ≈100 μm while creating a favorable niche for bacteria to thrive and spread.^[^ ^8^, ^9^ ^]^ Compared to the planktonic form, bacteria within biofilms show high resistance to common antimicrobials and host immune responses, primarily due to the protective EPS shield and confined arrangements and structure impeding the penetration of antimicrobials through the biofilm.^[^ ^9^, ^10^, ^11^ ^]^ This evasion mechanism perpetuates an increased inflammatory response and severely impedes tissue repair, rendering the wound unresponsive to conventional antimicrobials.^[^ ^12^ ^]^ As the conventional approaches fail to effectively overcome the formidable physical barriers of wound biofilms, this results in prolonged treatment and inevitable progression toward chronic wounds.^[^ ^13^ ^]^ Furthermore, the alarming rise in antibiotic‐resistant strains of bacteria has significantly limited the efficacy of conventional antimicrobial therapies necessitating a paradigm shift toward new and innovative solutions.^[^ ^14^ ^]^ Addressing these challenges demands a multifaceted approach, encompassing both novel delivery systems and treatment modalities for the management of chronic wound infections. Additionally, conventional antibiotic treatments often face limitations in achieving targeted and sustained effects, leading to suboptimal outcomes and potential resistance development.^[^ ^5^, ^15^ ^]^ As we navigate the evolving landscape of wound care and infectious diseases, the quest for new and innovative solutions becomes imperative.

Conventional and topical delivery methods have often faced limitations in effective infection control and tissue repair. Current clinical practices often rely on the application of topical creams, ointments, gels, and lotions containing antibiotics or antifungal agents in combination with a variety of silver dressings for the treatment of infected wounds.^[^ ^15^, ^16^ ^]^ Research efforts in the development of hydrogel delivery systems, nanoparticles (NPs), and other advanced materials have significantly improved the prospects of localized and targeted wound interventions.^[^ ^17^ ^]^ These approaches aim to enhance the precision, efficacy, and duration of treatment while minimizing the risk of systemic side effects. However, wound biofilms are far more challenging with layers of complexities depending on the location, duration, and wound status.^[^ ^18^ ^]^ Current approaches have shown great efficacy in the use of topical antimicrobials against superficial infections.^[^ ^19^ ^]^ However, the efficacy is severely impeded when faced with infected chronic wounds, particularly those harboring biofilms of polymicrobial bacterial and fungal species. Moreover, in chronic wounds, the presence of wound exudate creates a microenvironment rich with enzymes that can disrupt the efficacy of topically administered therapeutics.^[^ ^20^ ^]^ Importantly, biofilm‐associated wounds, distinguished by the resilient composition and structural organization in deeper wounds, present a formidable challenge for traditional treatments due to the refractory nature of antimicrobials.^[^ ^21^ ^]^ Topical application proves inadequate for deep cutaneous infections, hindered by limited drug penetration and the presence of substantial wound exudate. This inadequacy underscores challenges in maintaining sufficient antimicrobial concentration and functionality, necessitating frequent and high‐dose applications. Research efforts have therefore substantially recognized microneedles (MNs) as effective tools for drug delivery systems to address the challenges in the management of wound infection through localized delivery of antimicrobials offering simple, safe, and efficient techniques to increase drug delivery and bioavailability.^[^ ^22^, ^23^ ^]^

### 1.1 Overcoming Drug Delivery Barriers: The Vital Role of MNs

The human skin is a multilayered structure that primarily consists of the epidermis, dermis, and subcutaneous tissue.^[^ ^24^ ^]^ The epidermis has two layers, where the outer layer known as the stratum corneum (SC) acts as a barrier to transdermal penetration, with a unique arrangement of hydrophilic keratin proteins and packed hydrophobic lipids.^[^ ^25^ ^]^ Microbial infections breaching the skin barriers can impact one or more of the three skin layers, with a high susceptibility to migrate toward deeper layers for enhanced protection and habitation.^[^ ^26^ ^]^ Commensal and pathogenic microbes including bacteria and fungi colonizing the wound surface have been shown to lead to the development of clinical wound infection.^[^ ^27^ ^]^ While topical drugs effectively treat superficial infections, they struggle with deep cutaneous infections because of reduced drug permeability through the SC, leading to incomplete elimination of the infection source.^[^ ^28^ ^]^ Skin infections are challenging to treat systematically with antimicrobial agents due to low drug availability at the infection site, significantly reducing therapeutic impact. Similarly, the topical application of antimicrobials faces severe limitations in penetrating deep into the wound, resulting in low concentrations at the active site and poor antimicrobial efficacy.

To overcome this significant medical challenge, MN have gained increased attention as a potential solution by facilitating, targeted, and controlled drug delivery hence overcoming barriers posed by biofilms and the complex wound microenvironment. The concept of MNs was first reported in 1976 and aimed to overcome the major drawbacks of conventional transdermal drug delivery. Their minimally invasive and convenient properties can penetrate the SC of the epidermis without contacting nociceptive nerves and blood vessels.^[^ ^29^ ^]^ The SC typically with a thickness of (10–30 μm) presents a formidable barrier for therapeutic penetrations.^[^ ^30^ ^]^ The MN geometries can be tailored to penetrate the required depth of the skin barriers. This unique feature enables MNs to deliver drugs into the wound bed and even address bacterial biofilms in a minimally invasive manner. Given all these possible benefits, it is not surprising that there is widespread interest in MNs for various conditions. While MN design and development have been extensively researched and applied in medical areas including cancer treatment^[^ ^31^ ^]^ and vaccine delivery,^[^ ^32^ ^]^ there have been comparatively limited studies of the potential applications in addressing antimicrobial challenges faced during wound management. Researchers have responded to this challenge by developing MN patches for transdermal drug delivery to treat bacterial infections with tailored properties including physical disruption of bacterial biofilm, localized drug delivery directly to the active growth area of pathogenic bacteria, sustained delivery mechanism, biologically triggered antibacterial release system, and ability to diagnose and monitor changes in wound status (temperature, pH, infection) either locally or remotely.

**Figure 1:**
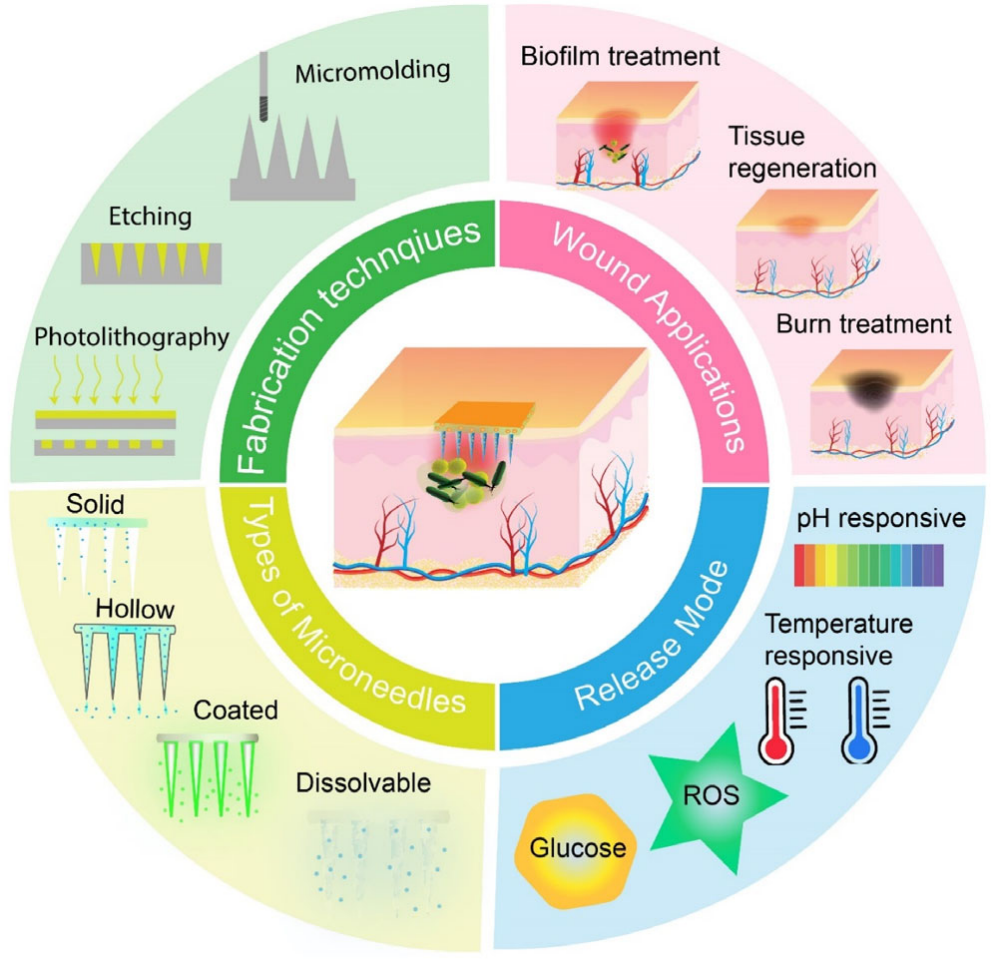
Schematic illustrations summarizing the design, development, delivery mode, and applications of MNs commonly used in transdermal wound applications. Developed MNs exhibit specific features tailored to address complex wound infections, employing either a passive or stimuli‐responsive system for a localized and secure antimicrobial delivery approach.

This comprehensive review described the current intricate landscape of skin‐mediated drug delivery, offering a detailed exploration of the skin's layers highlighting the pivotal roles in facilitating drug delivery during wound management. The article not only elucidates the structural and functional nuances of skin layers but also ventures into the diverse applications wherein skin‐mediated drug delivery proves instrumental. The review goes further to exemplify various delivery methods, showcasing commercially available products with clinical utility. Moreover, it addresses current research limitations, including formulation stability and regulatory considerations, and outlines prospects for the evolution of skin‐mediated drug delivery techniques for clinical wound management while critically discussing the advantages and disadvantages of current approaches (**Figure** **1**).

## 2 MN Technology

### 2.1 Overview of MN Design and Fabrication

Over the years, there has been substantial effort in identifying effective drug delivery strategies to address critical factors in the realm of healthcare. These efforts stem from the recognition that the efficacy and success of therapeutic interventions heavily rely not only on the drugs themselves but also on how efficiently and precisely drugs can be delivered to the intended target within the body.^[^ ^33^ ^]^ The evolution of MN design, fabrication, and application for wound management has fostered a promising modality.^[^ ^34^ ^]^ MNs, typically ranging from 10 to 2000 μm in length with a diameter of less than 300 μm, represent a minimally invasive, painless, and convenient solution to overcome many of the technical and delivery challenges of therapeutics.^[^ ^35^ ^]^ The height of the MNs can be readily adapted according to the specific applications. For example, transdermal antimicrobial delivery is much shorter while the penetration for blood extraction requires a depth of ≈1500 μm. The technology is unique and promising to ensure effective penetration of drugs into the skin in a painless manner using multiple micron‐scale needle arrays attached to a supporting membrane. During the past two decades, remarkable evolution has been witnessed in the development of MN‐based drug delivery systems targeting different organs, conditions, applications, and outcomes with greater emphasis on safety for broader clinical applications.^[^ ^36^ ^]^ The manufacturing method for MNs varies depending on the design, materials, and intended applications. Over the years, many materials have been utilized to manufacture MNs, including metal, glass, silicon, and different polymers either individually or in combination.^[^ ^37^ ^]^ The selection of materials and fabrication methods plays a crucial role in determining the translational potential of MNs. There have been various methods of fabrication including micromolding, lithography methods, 3D printing, microelectromechanical systems, chemical etching, laser ablation, and wet etching. The different types of MN fabrication are summarized in a recent review.^[^ ^38^, ^39^ ^]^ There's a significant focus on using reproducible fabrication techniques to tackle key application challenges, such as safety, drug loading, dissolution rate, ease of use, scalability, cost‐effectiveness, and long‐term stability.

### 2.2 Types of MNs for Antimicrobial Drug Delivery

Effective skin penetration and drug delivery of MNs are significantly influenced by the selection of materials and fabrication techniques aimed at enhancing MN structure and mechanical properties. The choice of materials, shape design, and manufacturing methods are essential to enhance MN penetration without inducing a negative immune response, ensuring comprehensive coverage of the affected wound area and maintaining widespread biocompatibility for clinical applications during wound management. Over the years, many different types of MNs have emerged all differentiated based on fabrication techniques, materials, layers of arrangement, and modes of drug delivery.^[^ ^40^ ^]^ Hence, MN types are categorized based on various factors, including manufacturing method, material composition, geometrical design, and application strategy.^[^ ^39^ ^]^ MNs come in diverse types including solid, hollow, coated, dissolvable, polymeric, metallic, and silicon MNs, all of which can be tailored to specific MN applications. The choice among these MN types depends on various factors including the specific drug or substance being delivered, targeted application, patient comfort, cost, and scalability associated with the MN fabrication.^[^ ^41^ ^]^ MNs can also be manufactured with different layers, where two‐layered MNs are becoming a current trend with increased drug loading and release of controlled strategy.

#### 2.2.1 Solid MNs

**Figure 2:**
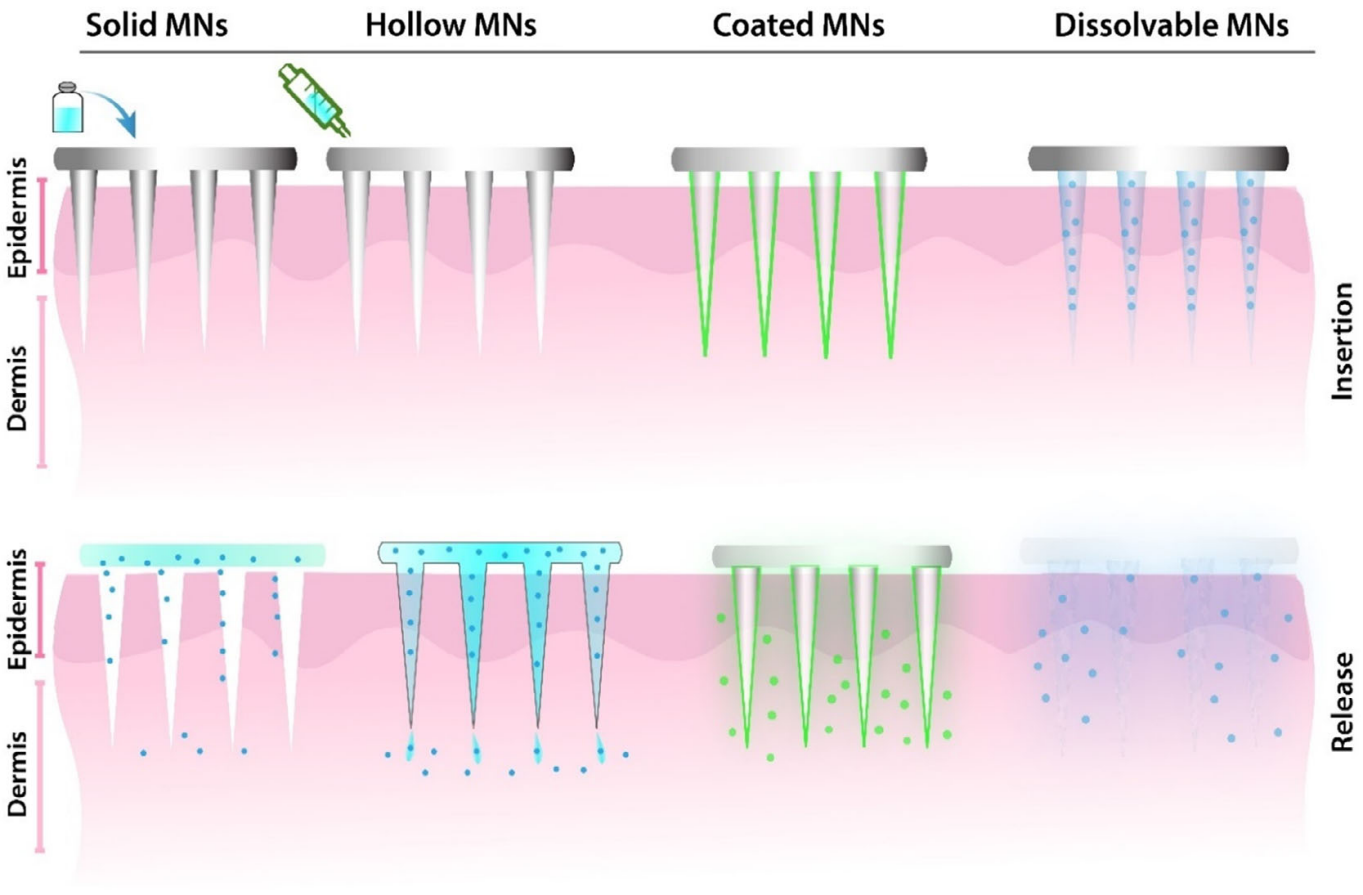
Schematics of different types of common MNs for transdermal delivery. The structures and expected outcomes of each of these MNs following insertion into the skin for localized drug delivery. Note: application in open chronic wounds also facilitates drug delivery deep into dermis‐targeting bacterial biofilms in deep wound beds.

Solid MNs are characterized by a nonporous structure without internal channels. This type of MNs was the first invention often consisting of silicon, metals, and polymers.^[^ ^42^ ^]^ These MNs are designed to create micropores in the SC, the outermost layer of the skin, facilitating enhanced permeation of drugs or therapeutic agents.^[^ ^43^ ^]^ The simplicity and effectiveness of solid MN facilitate suitability for a variety of applications where controlled drug delivery through the skin is desired. Solid MNs are typically used in conjunction with transdermal formulations including patches, lotions, creams, etc., as shown in **Figure** **2**. Solid MNs consist of a two‐step process based on the “poke‐and‐patch” approach where the microarrays create microchannels followed by the application of drug formulation permeating through passive diffusion across skin layers.^[^ ^44^ ^]^ Solid MNs are therefore highly useful delivery of larger molecules to permeate across the basement membrane. A recent study has used solid MN rollers to facilitate the diffusion of doxycycline across the skin which often is not feasible due to its high molecular weight and hydrophilicity.^[^ ^45^ ^]^ The application of doxycycline‐loaded MNs shows sufficient drug permeabilization required to inhibit key cellular features supporting tissue repair compared to controls. While solid MNs boast impressive mechanical properties, the risk arises if the needle breaks or sustains damage during application, potentially resulting in the needle body becoming lodged in the skin during drug delivery as summarized in **Table** **1**. This poses a significant safety hazard for the patient. Moreover, there are risks of infection associated with reuse and applications. There has been a great emphasis in the last few years on designing alternative needle geometries that can tolerate penetration forces without any deflection that can cause breakage.^[^ ^46^ ^]^ Solid MNs facilitate the permeation of drugs into a variety of tissue locations where the skin thickness varies depending on the location applied. A recent study has used an isotropic xenon difluoride (XeF_2_) dry etching process to fabricate silicon solid MNs using a photolithographic fabrication process.^[^ ^47^ ^]^ The MNs were designed with different geometries, numbers, and dimensions (length and height) enhancing functionality. The mechanical strength and performance of the MN were tested using both chicken and porcine skin offering a consistent pathway to deliver drugs at a desired skin location with uniform penetration and depth efficiency. Overall, solid MNs are most simple and often used for the permeation of therapeutics for different applications including cosmetics, vaccines, and general transdermal drug delivery. Most of the MNs employed in cosmetic industries rely on solid MN leveraging its mechanical properties and ease of fabrication.

**Table 1:** Summary of different types of MNs under development used for clinical wound management applications. Various MN designs have been proposed while the most common ones including solid, hollow, coated, and dissolvable microneedles have longstanding use for biomedical drug delivery applications. Depending on the intended application, each of these platforms has distinctive features that make them standout for wound infection. These diverse types of MNs, each with unique characteristics, play a crucial role in advancing drug delivery systems, offering innovative solutions for efficient therapeutic interventions.

Types	Applications	Common materials used for preparation	Advantages	Limitations	Reference
Solid MN	• Transdermal delivery • Cosmetic • Vaccine delivery • Interstitial fluid sampling • Blood/wound fluid extraction	Stainless steel, polylactic acid (PLA), polylactic*‐*co‐glycolic acid (PLGA), polycarbonate, copolymer, castable resin, polydimethylsiloxane (PDMS), silicon, glass, nickel, titanium	High mechanical strength Enhanced drug permeation Ease of fabrication	Passive release Prone to breakage within tissue Possibility of infection due to reuse Poor patient compliance	[38, 199]
Hollow MN	• Transdermal delivery • Vaccine delivery • Biosensing	PLGA, PLA, poly(methyl methacrylate), glass, nitinol, silicon, titanium, carboxymethyl cellulose (CMC), dextran	High loading capacity Flexible with small‐large drugs Constant flow rate Controlled dosage High drug bioavailability Suitability for the design of theragnostic tools for wound infection detection and treatment	Complex fabrications Drug leakage and clogging Safety issues Not self‐administrable	[200, 201]
Coated MN	• Cancer • Wounds • Diabetes • Antibacterial	Silicon, nickel, stainless steel, titanium, polyethylene glycol dimethacrylate (PEGDMA)	Small drug delivery Low drug loading efficiency High mechanical strength Self‐administrable Reduced dose frequency	Complex fabrication	[202]
Dissolvable MN	• Wound biofilm infection • Drug delivery • Vaccine applications • Cosmetics • Biosensing • Cancer • Regenerative medicine (cell therapy)	Hyaluronic acid, PLA, polyvinyl alcohol (PVA), polyvinylpyrrolidone (PVP), CMC, sucrose, fructose hydroxypropyl cellulose (HPC), carboxymethyl cellulose (CMC), polyethylene glycol (PEG), PLA, polyglycolic acid (PGA), chitosan, silk, chitin	High safety profile Controlled release kinetics Patient compliance Biodegradable Ease of fabrication Low‐cost production Self‐administrable One‐step application Noninvasive No residue	Mechanical strength Burst release Imprecise dosage Poor physical stability Limited loading efficiency	[93, 203, 204]

#### 2.2.2 Hollow MNs

Hollow MNs have interior space inside the needle, similar to micro‐syringes in that this MN design allows for the delivery of large amounts of drugs directly through the hollow core Figure 2. The opening tip of the needles allows the drugs to diffuse through and be delivered to the targeted region of the upper papillary dermis and subcutaneous tissue.^[^ ^48^ ^]^ Hollow MNs are versatile and can accommodate a range of therapeutic agents, including liquid drugs and NPs. Despite the similar action to subcutaneous injection, it has been shown that hollow MNs are more tolerable than subcutaneous injections because of the shorter needle length, which does not reach the nerves and therefore does not cause pain.^[^ ^49^ ^]^ The cavities of hollow MNs can store and continuously deliver drugs and also facilitate the collection and analysis of biological samples including tissue or wound fluid, blood, and cellular samples.^[^ ^50^ ^]^ However, the utilization of hollow MNs is constrained by the intricate fabrication and the process, which relies on a combination of multiple techniques including laser microfabrication,^[^ ^51^ ^]^ deep ion etching reaction,^[^ ^52^ ^]^ deep X‐ray photocopying, 3D printing,^[^ ^53^ ^]^ and wet chemical etching. This makes hollow MN fabrication complex and necessitates an experienced user while also contributing to a significantly higher product cost. Hollow MNs have however been shown to enhance penetration of drugs across the skin overcoming the use of permeation enhancer, which is commonly used in drug delivery, and this is a significant advantage of this MN design.

A recent study has developed a porous silicon MN having a tunable porous structure on the MN surface enabling increased loading efficiency of drugs.^[^ ^54^ ^]^ The type of hollow MNs shown to accommodate both small and macromolecular drugs that are uniformly loaded into the porous layer of the MN arrays. The study was validated using ex vivo penetration experiments on porcine tissue showed an efficient transdermal delivery with high mechanical strength. Another recent study by Liu et al. reports increased drug loading using hollow MNs with increased convenience of loading diverse drugs with tailored release kinetics.^[^ ^55^ ^]^ The MNs described consisted of three layers: an outer hydrophobic shell of polycaprolactone (PCL), an inner hydrogel shell comprising a combination of polyvinyl alcohol/chitosan/polyvinylpyrrolidone—PVA/CS/PVP, and a hollow cavity for loading the metformin diabetic drug. In this design, the outer hydrogel shell modulates core size for drug loading capacity, and the height of the middle hydrophobic shell can be adjusted for drug release kinetics. By combining MNs with different drug‐release kinetics on a single patch, multi‐timepoint drug delivery is achieved, enhancing efficacy and convenience.

Hollow MNs are also commonly used for electrochemical biosensors for interstitial fluid (ISF) or more importantly for glucose monitoring. This technology is designed with wearable options to provide reliable glucose information for diabetic patients.^[^ ^56^ ^]^ It is reported that MNs can provide in situ information or collect samples either for real‐time analysis or provide rapid samples for diagnostic purposes.^[^ ^57^ ^]^ This approach would also be favorable in wound management of infected diabetic ulcers allowing the use of hollow MNs for both glucose or infection monitoring and treatment simultaneously. Another study reports hollow MNs modified with conductive pastes and functionalized with CS cross‐linked with glutaraldehyde to provide the sensor with anti‐biofouling and adsorption of methotrexate (MTX).^[^ ^58^ ^]^ The in vitro and ex vivo characterizations showed that the functionalized hollow MNs are able to sense the therapeutic concentration of MTX and can also generate a closed‐loop system for MTX delivery. The reported MNs show a potential approach for effective sensing and delivery of anticancer drugs or therapeutics for the management of rheumatoid arthritis, wound infection, or psoriasis.

#### 2.2.3 Coated MNs

Coated MN platforms coat or encapsulate drug formulations onto MN film to form film cores as shown in Figure 2. The MNs are fabricated using either metals, silicon, or polymers. This system serves as a one‐time delivery method, employing a dip‐coating or spraying process to coat both hydrophilic and hydrophobic drugs onto solid MN surfaces. The drug release profiles heavily rely on the physicochemical properties of the coating formulations and the interactions between the coating layer and the MN substrate. After the MNs enter the skin, the painted or encapsulated film dissolves and penetrates the tissue as intended. The coating may be tailored to release the drug over a specific period, offering precision in therapeutic delivery. Coated MNs are advantageous for applications requiring targeted and sustained drug release. Coated MNs are excellent for painkillers that usually produce pharmacological effects immediately in small doses or for steroids to provide anti‐inflammatory and analgesic effects. The amount of drug loaded onto the MNs is typically determined by the thickness of the coating that is applied to the surface of the MNs by dipping, spraying, or coating.^[^ ^59^ ^]^ To generate a flexible and reproducible coating, dip‐coating, spray coating, and inkjet printing methods have been developed in recent years to generate precision drug coatings to ensure a high portion of the coated therapeutic dose is delivered to the target applications.^[^ ^60^ ^]^ The coated MNs allow better flexibility with the coating to control the release. A study has generated porous pH‐responsive polymer‐coated MNs that enable the pH‐responsive release of the model drug.^[^ ^61^ ^]^ A study reported the fabrication of poly(ethylene glycol) diacrylate MNs coated with a blend of gelatin and sucrose film on the surface. This study used the casting drying process to ensure uniform film formation. The study demonstrated successful delivery of bovine serum albumin and doxorubicin drug encapsulated in the film to validate release and penetration using ex vivo models. The coated MNs showed good mechanical strength, enabling efficient skin insertion without any major deformation.^[^ ^62^ ^]^ Coated MNs are less commonly employed due to the poor loading capacity of the drugs. The advantages and limitations of this type of MN are summarized in Table 1. The drug loading capacity of coated MN is limited by the specific surface area of MNs often being insufficient to load higher amounts for long‐lasting applications. Another limitation reported is the potential loss of drugs during the fabrication process; hence, alternative coating and fabrication strategies are explored to maintain the drug deposition.^[^ ^63^ ^]^

#### 2.2.4 Dissolvable or Biodegradable MNs

Dissolvable MNs are developed using water‐soluble or biodegradable polymers and it is the most utilized system for biological use. Dissolvable MNs are often fabricated using micromolding, laser lithography, photolithography, and 3D printing.^[^ ^64^ ^]^ Among all, micromolding is the most prevalent and economical choice, offering a streamlined alternative to complex, multistep processes.^[^ ^65^ ^]^ The process is simply achieved by blending the polymer solution into the female mold, followed by the solidifying and demolding process. The dissolvable MNs are simplified into one‐step administration, where the MNs pierce the skin to release cargo without additional manipulation. Upon skin insertion, these MNs dissolve in contact with the wound fluid or other biologics facilitating a gradual release of loaded drug molecules overtime as shown in Figure 2.^[^ ^66^ ^]^ Depending on the polymers utilized, it usually disintegrates completely offering high patient compliance and leaving no biohazardous waste.^[^ ^67^ ^]^ This feature enhances the convenience of self‐administered therapy, offering an efficient and eco‐friendly approach to drug delivery. The concept of dissolvable MNs has also expanded into hydrogel‐forming MNs, which are fabricated from polymeric materials that have been cross‐linked.^[^ ^68^ ^]^ This kind of MN array draws up ISF, causing the polymeric matrix to swell allowing diffusion of drug substance through the swollen matrix into the dermal tissue. A large selection of biocompatible and water‐soluble polymer materials with multiple properties can be used to fabricate dissolving MNs as summarized in Table 1. A wide range of dissolvable polymers used for MNs are greatly reviewed for drug delivery.^[^ ^69^ ^]^ The composition of the dissolvable polymers can be adjusted and modified to enable desirable targeted drug delivery. A study utilized doxycycline‐loaded NPs in dissolvable MN made of PVP and PVA, showing improved biofilm penetration and bacterial infection targeting.^[^ ^70^ ^]^ These MNs demonstrated superior release of doxycycline in response to bacteria, with better dermato‐pharmacokinetic profiles compared to patches without MNs. The study also revealed a 97% reduction in biofilm bioburden after 48 h, indicating a high release rate and efficacy against deep infection.

Recent studies have also reported that dissolving MNs are usually a fast‐dissolving system after being inserted into the skin and often encounter challenges related to poor skin penetration, attributed to the mechanical limitations of the water‐soluble materials.^[^ ^71^ ^]^ This aspect may not be ideal for drugs necessitating sustained and long‐term applications for example in the healing of chronic infected wounds. Achieving a delicate balance in dissolution is crucial to ensure the complete and gradual release of therapeutics, thereby reducing the need for frequent changes. Thus, complete drug delivery is more likely to be impeded, leading to medication wastage, imprecise dose, and limited drug delivery efficiency. Incomplete insertion of MNs has been openly highlighted as a significant problem in using dissolvable MNs. A recent study has designed dissolvable MNs analyzing different geometrical parameters to ensure that the MN is inserted efficiently into the skin.^[^ ^72^ ^]^ The research compared three common shapes of MN: conical, funnel, and candlelit while investigating their insertion depth and efficacy. The characterization unveiled a strong correlation between MN shape and insertion depth. Specifically, the candlelight design demonstrated significantly enhanced drug delivery efficacy, as evidenced by insulin delivery tests conducted on the skin of C57BL/6 (a common inbred strain of laboratory mice) mice. This study establishes an important criterion for selecting the optimal shape and design to maximize penetration and volume delivery across the different biological membranes.

Research into penetration efficiency is ongoing, with studies exploring various strategies to enhance permeation and enable precise drug release. The aim is to develop MNs that can dissolve or allow the removal of backing layers, providing flexibility and adaptability in their application. Separable MNs are becoming an interesting area of research development. A recent study reports a rapidly separating dissolving MNs system, which was designed to rapidly detach from the drug‐concentrated tip when inserted into the skin allowing accurate dose release of therapeutics.^[^ ^73^ ^]^ The separating part aids in overcoming skin deformation, and dissolves quickly by the skin's ISF, enabling effective detachment from the MN base. The base of the dissolvable MN was fabricated using complexes of polymer including sugars with different ratios to enhance mechanical performance and disintegration rate. The disintegration and penetration were validated using an ex vivo permeation study while the model drug of diclofenac sodium was used for an in vivo pharmacodynamic.

Dissolvable MNs are also commonly employed for wound application. Long et al. reported hyaluronic acid dissolvable MNs loaded with poly(lactic*‐*co‐glycolic acid) (PLGA) NPs to insert the skin efficiently while releasing recombinant humanized collagen type III (rhCol III).^[^ ^74^ ^]^ The study demonstrated improved skin cell function including cell proliferation and migration. In a preclinical mouse model of diabetic wounds, the MN successfully traversed the tissue barrier of skin necrosis without causing collateral tissue damage, leading to enhanced wound closure post‐application. Another study used PVP dissolvable MNs to deliver antimicrobial peptides (AMP) alongside a photothermal conversion agent.^[^ ^75^ ^]^ Upon exposure to near‐infrared (NIR) light, the MNs underwent phase changes, releasing the antimicrobial agent and enhancing biofilm penetration for effective infection elimination. The photothermal modalities also generated heat, aiding in MN dissolution without any toxicity to the animal. Additionally, Su et al. used a similar approach and reported a dissolvable PVP–MN designed as a nanofiber dressing loaded with AMPs to eradicate biofilm infection.^[^ ^76^ ^]^ The MN featured a W379 (database‐designed peptide with eight amino acids [sequence: RRRWWWWV]) in‐house‐made AMP with a similar structure to the human cathelicidin (LL‐37) AMP, incorporated into electrospun nanofibers. Upon application, the MN was dissolved within 3 min to disintegrate the peptide allowing uniform diffusion. The study reports that MN array aids in enhancing peptide penetration for improved efficacy compared to free drugs and this approach was validated by being able to eradicate Methicililin‐resistant Staphylococcus auresu (*MRSA)* bacterial biofilm in a diabetic defective mouse (000697‐B6.BKS(D)‐Leprdb/J [mice homozygous for the diabetes spontaneous mutation]) model suggesting that this treatment provides an effective strategy to downregulate chronic wound biofilms.

There is a clear indication that dissolvable MN are frequently chosen for fabrication due to their ease of preparation and the abundance of biodegradable polymers. These polymers can dissolve either in interstitial or wound fluid or in response to the surrounding microenvironment, including changes in pH or enzyme present in the microenvironment, leading to complete dissolution without leaving residues. This approach avoids the risk of biological hazards or waste, making this design suitable for clinical applications. Furthermore, dissolvable MNs offer a high degree of versatility in biomedical applications. The ability to tailor MNs to dissolve in response to specific physiological conditions makes this approach highly adaptable for various drug delivery scenarios.

## 3 Recent Advances in MN‐Based Drug Delivery Systems

Recent advances in MNs application have marked a transformative phase in the field of healthcare. MNs are a versatile platform, offering a minimally invasive approach for efficient drug delivery, diagnosis, and monitoring. It has enabled more targeted and controlled release of drugs, vaccines, and therapeutic agents, enhanced treatment efficacy while minimizing side effects. The continuous evolution of MN‐based drug delivery systems underscores the potential of MNs to revolutionize the landscape of pharmaceutical interventions for all wound management, cosmetic, and pharmaceutical industries. The treatment of wound infection is a complex process requiring often a multifaceted approach with precision drug delivery to not only control bacterial infections but also prevent infection recurrence and support wound healing and tissue regeneration.^[^ ^77^ ^]^ Hence, MNs have been gaining strong attention for wound management with a strong emphasis on smart drug delivery systems, ushering in a new era of precision and efficiency in wound interventions, enabling targeted and controlled release of therapeutic agents. Smart MNs involve the integration of sensors and responsive materials, allowing for real‐time monitoring and adjustments based on the physiological wound microenvironment. This is anticipated to overcome many of the current challenges of traditional wound therapies, improving current treatment outcomes. Therefore, the intelligent and patient‐centric approach of smart drug delivery using MN for wound infections represents an innovative avenue for personalized medicine and the future of healthcare.

### 3.1 The Clinical Challenges of Wound Infections and the Role of MNs

Wound healing is a highly coordinated, multistep process that occurs in overlapping phases of hemostasis, inflammation, proliferation, and remodeling resulting in tissue repair and regeneration.^[^ ^77^, ^78^ ^]^ Generally, wounds are categorized either into acute or chronic depending on the wound severity and the time taken to heal. Acute wounds generally proceed through the phases of healing in a timely and coordinated manner. Unlike acute wounds, chronic wounds are characterized by a prolonged and impaired healing process, where the healing persists for up to 3 months or longer.^[^ ^79^ ^]^ Certain wounds may fail to heal completely, leading to significant complications including nontraumatic amputations, increased morbidity and mortality rates, and recurrent complexities.^[^ ^80^ ^]^ These wounds, often associated with underlying health conditions such as diabetes, vascular disorders, or persistent infections, deviate. The disrupted healing cascade in chronic wounds necessitates specialized and comprehensive clinical care, requiring repeated treatment and care.^[^ ^81^ ^]^ The management of infections is often treated with topical antimicrobial agents and debridement to remove necrotic tissue while the use of specialized dressing is used to support the healing process. A variety of hydrogels have been optimized to not only deliver antimicrobials but also stimulate the healing process. Some degree of success has been observed when the wounds are at an early stage using advanced delivery systems using silver NPs (AgNPs),^[^ ^82^ ^]^ AMPs,^[^ ^83^ ^]^ 2D antimicrobial materials like black phosphorus,^[^ ^84^ ^]^ or photothermal‐activated therapies.^[^ ^85^ ^]^ Research to date has shown that scaffolds have also been popular as a means of sustained delivery systems while modulating chronic wound conditions to target biofilm and improve healing.^[^ ^86^, ^87^ ^]^ Despite considerable efforts in developing various therapeutic products, the efficacy of conventional treatments has been limited to satisfy the effective clinical management of infected chronic wounds.

**Figure 3:**
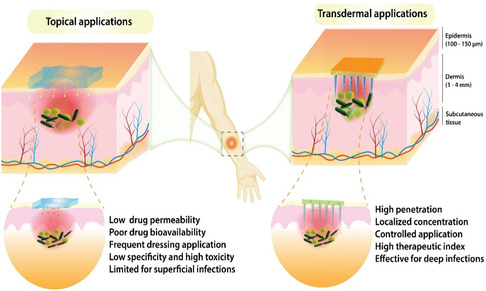
Schematic illustration showing the comparative penetration depths of topically applied medications versus transdermal MN applications. Topical application involves the direct application of medication onto the skin surface, with penetration limited to the epidermal layer and superficial upper papillary dermis. In contrast, MN applications puncture the skin's outer barrier, facilitating deeper penetration of drugs into the dermal layers toward subcutaneous tissue.

A recent review summarizes the development of various types of antibacterial dressings.^[^ ^88^ ^]^ Overtime, the efficacy of antibacterial dressings or antibiotics has fluctuated in their ability to diminish the initial onset of clinical infection. Despite the continuous research and development in various types of dressing, there is a major gap associated with addressing chronic deep infections.^[^ ^89^ ^]^ As a result, the current dressings are used repeatedly at higher doses to attain the necessary therapeutic bioavailability. In many instances, major challenges arise whereby antibiotics may prove ineffective in reaching the affected site at optimal concentrations or, conversely, be excessively delivered, leading to potential toxicity and adverse effects.^[^ ^90^ ^]^ In the wake of the post‐antibiotic era, there's a growing focus on interdisciplinary research aimed at creating novel antimicrobials by harnessing the potential of nanotechnology. These efforts aim to create specialized and patient‐centric antimicrobial solutions using advanced delivery mechanisms against AMR and for effective wound management. There is a growing acknowledgment that localized, direct delivery of antimicrobials plays a crucial role in enhancing treatment outcomes and safety.^[^ ^91^ ^]^ Hence, different types of MN systems have been developed to precisely deliver antimicrobials to targeted sites, mitigating issues related to systemic exposure and enhancing the efficacy of treatment while minimizing the risk of adverse effects.^[^ ^92^ ^]^ The concept of topical treatment against transdermal antimicrobial application for wound management is summarized in **Figure** **3**, showing the distinct difference in drug deliver penetration pathway across the skin barriers to reach the infections that are deeply embedded in the papillary dermis or subcutaneous tissue of chronically infected wounds (Figure 3). Additionally, the growing interest in MNs arises for wound management due to the ability to address important parameters of wound care including the ability to diagnose and monitor wound status, restore skin hemostasis, and actively participate in wound sterilization and healing responses required for tissue repair and regeneration. These may not be possible with conventional systems. Delivery of antimicrobials using MNs has been widely studied because of the flexible properties enabling many types of drugs, NPs, peptides, growth factors, and cells. For example, MNs have been used to deliver mesenchymal stem cells (MSCs) with high cell viability, addressing a significant challenge in MSC delivery.^[^ ^93^ ^]^ This addresses the inherent instability and low cell viability of MSCs in traditional delivery methods. Given the challenges of infected wounds in a chronic state, the latest progress in MN technology has been proposed to improve the antimicrobial permeability, stability, and bioavailability of drugs or other therapeutics delivered directly to deep regions. This development enables one to transcend the physical barriers of the wounds (SC, wound exudate) and reach the affected site of biofilms presenting a promising solution for chronic wound management.

### 3.2 Overcoming Biofilm Infections through MN Innovation

Biofilm is a complex microbial community characterized by a protective matrix of EPS, protecting the bacteria from threats including harsh environmental conditions, antibiotics, bacteriophages, and the host immune response.^[^ ^94^ ^]^ Biofilms play a significant role in wound prognosis impeding the normal wound healing process, leading to chronic conditions.^[^ ^95^ ^]^ Chronic wounds provide an ideal environment for continuous microorganism growth due to several factors including persistent inflammation, impaired immune response, nutrient‐rich environment, and the presence of necrotic tissue and ample nutrient sources.^[^ ^96^ ^]^ These conditions create a moist and conducive growth condition that promotes the proliferation of bacteria and other pathogens therefore driving biofilm formation often underpinning the development of clinical infection and sepsis. The protective nature of the biofilm matrix makes it challenging for traditional antibiotics to penetrate and eradicate the bacteria effectively.^[^ ^97^ ^]^ In chronic wounds, the presence of eschar, discharge of exudate, and a harsh chemical microenvironment enriched with various enzymes disrupt the delivery of topically administrated therapeutics.^[^ ^98^ ^]^ The composition and organization of biofilms confer a natural barrier to the diffusion of antibacterial agents, leading to increased antibiotic tolerance and resistance development. Additionally, bacterial elimination during wound management is highly dependent on the dose of antimicrobials, and duration of action thereby emphasizing the important role of direct and localized antimicrobial delivery to enhance bioavailability and accumulation at concentrations required to fight against complex often polymicrobial infections observed in clinical wounds.^[^ ^11^ ^]^ Therefore, MN systems have recently been implemented to increase the availability of therapeutics within the wound bed with a controlled spatial distribution.

**Figure 4:**
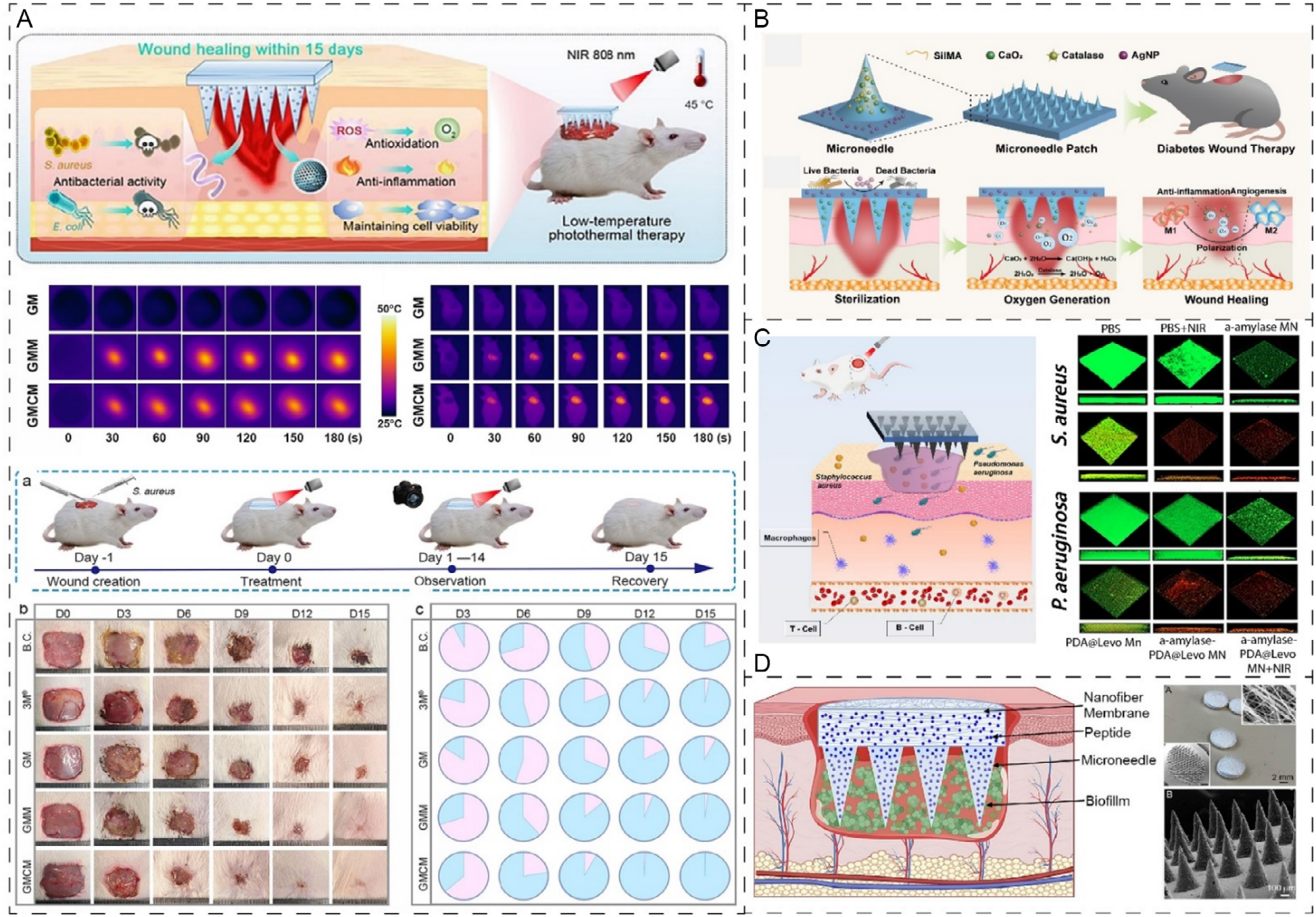
A) The development of NIR‐responsive antibacterial GelMA microneedles infused with antimicrobial peptides with a dual antibacterial mechanism to eradicate *S. aureus* bacterial chronic infection and support wound healing after 15 days of application. Adapted with permission.^[^ ^99^ ^]^ Copyright 2024, Elsevier. B) Development of oxygen‐releasing and antibacterial AgNP to maintain sterility and accelerate diabetic wound healing. Reproduced with permission.^[^ ^102^ ^]^ Copyright 2024, Elsevier. C) Elimination of bacterial biofilm using NIR MN‐mediated bacteria killing through biofilm EPS breakdown, overcoming biofilm barriers to deliver levofloxacin. Reproduced with permission.^[^ ^103^ ^]^ Copyright 2022, Elsevier. D) Reports of Janus type dissolvable MNs arrays for effective delivery of database designed AMPs with tailored MNs arrays to target deep biofilms of MRSA. Reproduced with permission.^[^ ^76^ ^]^ Copyright 2020, American Chemical Society.

The innovation of antimicrobial MNs is gaining enormous ground using strategies to target biofilm structure through physical, chemical, or compositional means. For instance, there has been significant interest in stimulus‐responsive MNs with enhanced loading capacity, specifically designed for chronic wounds. A recent study used methacrylated gelatin (GelMA) MNs containing AMP assembled with manganese oxide NP to form a hybrid NP for photothermal antibacterial activity **(** **Figure** **4**A).^[^ ^99^ ^]^ The NPs were immobilized into the MNs and evaluated for photoresponsive antibacterial properties mediated by reactive oxygen species (ROS) scavenging and killing efficiency. As shown in Figure 4A, the NIR‐responsive MN was able to show excellent dual antibacterial activity to serve as a multifunctional dressing for controlling *Staphylococcus aureus* chronic wound infection after 15 days of repeated application. Su and colleagues have used AMP (W379) and monoclonal antibodies PBP2a within dissolvable MN as a selective strategy to target bacterial membranes showing exceptional efficacy in reducing bacterial burden.^[^ ^100^ ^]^ The PVP MN was evaluated using a Type II diabetic mouse wound model with *S. aureus* biofilm, demonstrating strong biofilm reduction capacity. After 48 h of application, it effectively eradicated the biofilm from the murine wounds.

MNs with oxygen‐responsive antibacterial properties have also been engineered to target the unique microenvironment of chronic wounds, addressing challenges associated with low tissue oxygenation and subsequent effects on healing.^[^ ^101^ ^]^ Sun et al. proposed the use of a dissolvable MN encapsulated with calcium peroxide (CPO), catalase, and AgNPs to continuously release oxygen, accelerating diabetic wound healing through effects on cellular function including proliferation and migration.^[^ ^102^ ^]^ The slowly released oxygen from the MNs relieves the hypoxic state of the diabetic wound tissues, restoring disrupted cellular and immune system functions and revitalizing blood vessels. Additionally, the MN was also shown to be effective in maintaining wound sterility and supporting wound healing over 12 day application (Figure 4B). Other strategies were also explored using MNs containing NPs capable of generating photothermal therapy (PTT) and photodynamic therapy (Figure 4C). Exposure to NIR irradiation can effectively eliminate bacterial biofilm structures. Given the challenge of biofilm structure, studies have used MNs to target the EPS matrix by enzymes α‐amylase to break down the EPS barriers and deliver the antibiotics more effectively within biofilm sites when exposed to PTT to eliminate infection and also accelerate the healing response after 11 days of treatment.^[^ ^103^ ^]^

The destruction of biofilm protective layers has been a major center of attention. Recently, a unique MN patch has been developed that can physically enter the biofilm matrix and deliver quorum‐sensing inhibitor luteolin (Le) to impair biofilm activities.^[^ ^104^ ^]^ The system was also supported by a nanomotor loaded with photosensitizer and nitric oxide (NO) donor which under NIR was able to enhance bacteria killing, drug penetration and support wound healing validated using in vitro and in vivo wound infection models. Su et al. designed an MN patch using a spun nanofiber membrane as the substrate and a tip loaded with AMPs, which could effectively remove biofilms, and support healing (Figure 4D).^[^ ^76^ ^]^ Meanwhile, Woodhouse and colleagues used a similar approach to develop a degradable MN patch containing PVP loaded with CPO with a polyethylene terephthalate (PET) substrate.^[^ ^105^ ^]^ Upon exposure to the wound, the MNs underwent degradation, releasing CPO, which subsequently decomposed into hydrogen peroxide and oxygen. This process effectively killed bacteria, as evidenced by biofilm culture testing. This proof‐of‐concept study was also validated using an ex vivo porcine model showing uniform penetration and killing offering innovative therapy; however, further research is required to demonstrate safety and efficacy in large animal preclinical wound infection models.

At the same time, there is ongoing debate surrounding the potential harm of using NIR, with concerns raised about its tendency to induce spatial un‐specificity and heat‐related damage to tissues.^[^ ^106^ ^]^ As an alternative, He et al. developed magnetothermal‐responsive dual‐layered MNs that avoid the use of NIR‐induced activity.^[^ ^107^ ^]^ This approach is designed to provide high precision in sterilizing wounds. Moreover, AgNP is widely used for antibacterial applications due to its excellent antimicrobial activity without the use of light, heat, or any external mechanisms. Gonzalez Garcia and colleagues used AgNPs to develop self‐sterilizing dissolvable MNs capable of suppressing bacterial pathogens as a potential wound patch.^[^ ^108^ ^]^ Similarly, AgNPs are also prepared in situ using polysaccharide MN offering ease of preparation, high safety profile, and strong anti‐biofilm properties to control bacterial wound infections.^[^ ^109^ ^]^ Hence, the utilization of AgNPs‐incorporated MNs is rapidly gaining momentum for application in wound management, owing to the ease of preparation, and its remarkable antibacterial efficacy against a broad spectrum of pathogens implicated in nosocomial infections.^[^ ^110^, ^111^, ^112^, ^113^ ^]^

Impregnating or coating AgNP in an MN matrix to control the release of silver ions and avoid direct exposure to healthy tissues is a viable strategy for preventing the accumulation of silver and reducing toxicity to mammalian cells. Currently, AgNPs are commonly used for localized delivery using hydrogel dressings.^[^ ^114^, ^115^, ^116^ ^]^ However, hydrogel applications are limited to superficial infections in wound management. Zhao et al. used MN patches to deliver combination therapy using AgNPs, glucose oxidase nanocapsules (nGOx), and apramycin utilizing antibiotics and non‐antibiotic treatment.^[^ ^117^ ^]^ Once delivered to the site of wound infection, nGOx can convert tissue glucose into hydrogen peroxide (H_2_O_2_), which in turn accelerates the erosion of AgNPs into Ag^+^, giving rise to a synergistic complementary cocktail of antibiotics and metal ions as a therapeutic effect. Using in vitro murine models, the study showed rapid bacterial killing at a relatively low antibiotic dose, which was further validated using topically applied MNs against both rabbit and mouse models with scarless skin recovery demonstrating favorable outcomes.

Burns are also a major clinical challenge as a result of deep tissue damage with severe burns often impacting subcutaneous tissue, susceptible to biofilms, and uncontrolled tissue scaring.^[^ ^118^ ^]^ Removing infection and modulating immune response have been the key to the application of MNs for burn tissue injury.^[^ ^119^ ^]^ MNs have been crucial in mitigating various inflammatory factors and promoting the phase transitions from inflammation to proliferation while inhibiting scar formation.^[^ ^120^ ^]^ Therefore, research efforts have made significant strides to tackle biofilms using innovative forms of MNs all tailored to address major gaps in wound management including wound sterilization, inflammation, and proliferation. These achievements resonate with the development and design efforts aimed at enhancing drug permeation and targeting key structural features of biofilms, including the bacterial membrane, EPS, DNA, and other vital biological mechanisms. These MN designs employ a combination of killing mechanisms, integrating antimicrobial release and PTTs to efficiently eradicate complex biofilms. In recent years, the research direction has shifted toward smart release MN systems integrated with biological sensors and monitoring devices to monitor the physiological parameters of the wound in real time, providing information about the wound healing process to assist in diagnosis and treatment adjustment.

### 3.3 MN for Smart Antibacterial Drug Release

Traditional MN drug delivery relies on passive drug release mechanisms, often leading to suboptimal therapeutic effects or the potential risk of drug overdose due to a lack of responsiveness to physiological conditions. In contrast, recent advancements in smart‐responsive systems have revolutionized drug delivery by providing precise and on‐demand release.^[^ ^121^ ^]^ Hydrogel has been a very popular drug delivery system due to its attractive features including creating a moist wound environment, absorbing exudate, and ease of functionalization to deliver payload based on various triggered stimuli (pH, temperature, etc.).^[^ ^122^, ^123^ ^]^ However, MNs are significantly more advanced, offering enhanced tissue penetration to the deep wound bed, a higher degree of drug loading, and better biofilm targeting.^[^ ^124^ ^]^ In particular, the use of smart MNs aims to mitigate adverse effects associated with off‐target therapies. Over the years, a variety of stimulus‐responsive MN have been designed to programmatically release drugs based on pathological characteristics or specific exogenous physical/chemical signals.^[^ ^125^ ^]^ These smart MNs are crafted from polymeric materials capable of undergoing swelling, disintegration, or dissolution in response to stimuli, facilitating the controlled release of therapeutics. Over the past decade, there has been a substantial increase in publications focusing on stimuli‐responsive MNs, targeting key parameters in the wound microenvironment including pH, temperature, enzymes, glucose, etc.^[^ ^126^ ^]^ For example, in addition to extrinsic factors, a bacterial‐responsive strategy has also been explored to target bacterial lipase resulting in MN component breakdown and therapeutic release.^[^ ^127^ ^]^ Other strategies are focused on microenvironmental factors detecting changes between healthy and pathogenic conditions.

#### 3.3.1 Thermoresponsive MNs for Wound Infection

Temperature stands out as a pivotal role in distinguishing between healthy and infected wounds. The skin temperature typically ranges from 33 to 37 °C; however, this temperature is easily altered in response to pathological wound infection conditions.^[^ ^128^ ^]^ The temperature of infected wounds typically is 3–5 °C higher than the surrounding healthy tissue, primarily because of escalated biochemical reactions, inflammation, and bacterial proliferation.^[^ ^129^ ^]^ A growing number of studies have used this mechanism as a key stimulus to deliver drugs via temperature‐responsive MNs.

**Figure 5:**
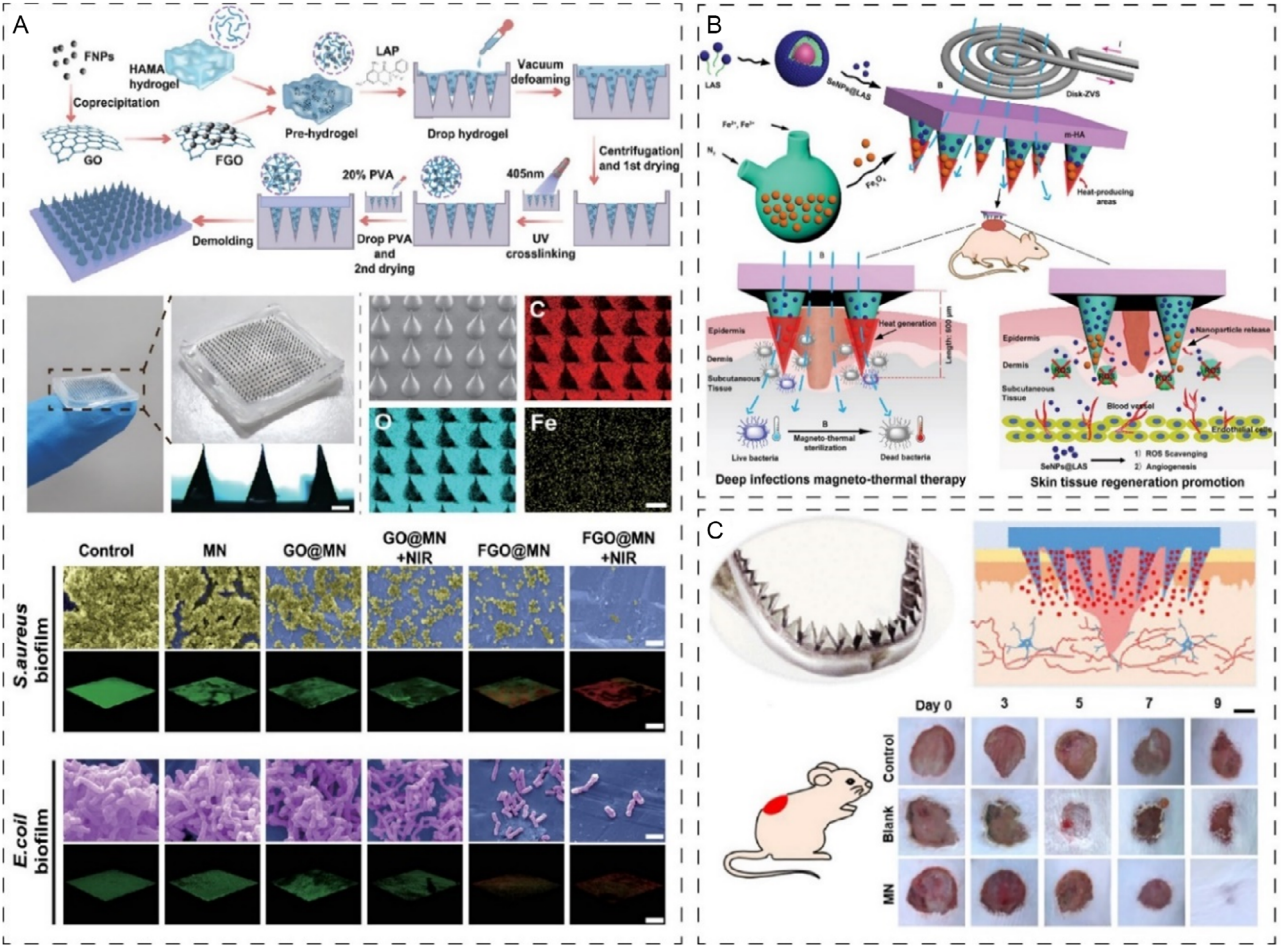
A) The generation and application of a photothermal methacrylated hyaluronic acid microneedle patch containing Fe_3_O_4_ nanoparticles on graphene oxide nanosheets. The temperature‐responsive MNs showed great localization and suppression of biofilm against *S. aureus* and *E. coli* biofilm while supporting the immune system to drive wound healing. Reproduced with permission.^[^ ^130^ ^]^ Copyright 2022, Wiley. B) Magnetothermal‐responsive MNs supplemented with an electromagnetic field disk to deliver antimicrobials deeply penetrating wound barriers and biofilm through hyperthermia sterilization. Reproduced with permission.^[^ ^107^ ^]^ Copyright 2024, Wiley. C) A prototype of shark‐teeth‐inspired MN dressing fabrication for the treatment of bacterial infection through temperature temperature‐responsive release mechanism. Reproduced with permission.^[^ ^131^ ^]^ Copyright 2021, American Chemical Society.

For example, Zhu and colleagues describe the photothermal MN patch to regulate the biofilm microenvironment.^[^ ^130^ ^]^ As shown in **Figure** **5**A, a photothermal MN patch is synthesized by the growth of Fe_3_O_4_ NPs on graphene oxide nanosheets and then encapsulated in methacrylated hyaluronic acid needle tips. The study reported that iron interference within the biofilm environment suppressed biofilm function against *S. aureus* and *Escherichia coli* while rejuvenating the immune system after 15 days of application. A similar study also reported the magnetothermal responsiveness using ferric oxide NPs (Figure 5B).^[^ ^107^ ^]^ He et al. developed magnetothermal‐responsive dual‐layered MNs functionalized with ferro ferric oxide with selenium NPs (SeNPs) alongside an electromagnetic field disk. The simulation showed high precision in penetrating the wound barriers and biofilm to perform targeted hyperthermia sterilization. The advantage of this approach included a gradual degradation of the needle and therefore controlled release of the SeNPs to reduce ROS and alleviate the wound inflammation, which has been demonstrated to regulate wound redox homeostasis and promote angiogenesis in diabetic murine models of wound infection. Hence, these versatile MNs can effectively reach deep infections, and offer noninvasive tissue penetration, and anti‐inflammatory effects, while simultaneously promoting angiogenesis offering a promising clinical potential for wound infections. Further validation of this approach in large animal models or clinical trials is pending.

A different study was inspired by the structure of shark teeth to develop a biomimetic MN for controlled release using a temperature‐responsive mechanism (Figure 5C).^[^ ^131^ ^]^ The hydrogel MNs were designed with arrangements similar to typical shark teeth, enhancing penetration capability and drug release at the physiological temperature of 37 °C. The diabetic animal wound experiments using human epidermal growth factor (hEGF) showed improved wound recovery serving as a smart release system to treat wounds within 9 days of application with a high degree of flexibility that could be applied to all kinds of wound interfaces. NIR‐responsive MNs generating localized heat have also been reported. For example, Fan et al. have shown that when the MN patch is composed of black phosphorus and gelatin, the NIR exposure can transform light into heat, thereby increasing the local area temperature and phase transition resulting in the detachment of the MN backing layer by shear forces.^[^ ^132^ ^]^ Subsequently, the MN can facilitate a sustained drug release due to the localized temperature change allowing black phosphorus to exert antimicrobial effects. However, the use of NIR and its complicated process has intrigued researchers toward a simpler and safer approach for clinical applications. This is often achieved using materials with thermoresponsive properties and higher biocompatibility. Li and colleagues grafted poly(N‐isopropyl acrylamide (Pnipam) onto gelatin to fabricate gelatin‐g‐Pnipam.^[^ ^133^ ^]^ This combination can impart sol–gel transition properties triggered by the changes in local temperature serving as a controlled drug carrier. In another study, Pnipam has also been used for temperature‐responsive release of insulin for diabetic murine wounds.^[^ ^134^ ^]^ In addition to wound infection, temperature‐responsive MN delivery has also been popular for cancer treatment, diabetes, and skin conditions using a variety of approaches to control the release, swelling, degradation, and stability for precise treatments.

#### 3.3.2 pH‐Responsive MNs

pH plays an important role in the cellular processes of wound healing and the wound microenvironmental pH is constantly and dynamically changing during tissue injury and clinical infection development. The normal pH of healthy skin is weakly acidic pH 5.5–pH 6.5, but wounds that are colonized with bacteria and develop infection show an elevated pH of above 7 and can reach up to pH 9.6.^[^ ^123^, ^135^ ^]^ The alkaline nature of the wound is shown to further deteriorate and delay the wound healing with susceptibility to further bacterial colonization and growth.^[^ ^136^ ^]^ Hence, changes in wound pH have been explored as the stimulus for designing pH‐sensitive drug delivery systems. Studies have used wound pH as a means of monitoring the wound status as an early indicator of infection risk.^[^ ^137^, ^138^ ^]^ For example, MNs can be designed as pH‐responsive carriers facilitating a self‐regulated local delivery to bacterial wound infection through a feedback loop strategy. This also has practical relevance in cancer therapy because the microenvironment of most tumors has a lower pH than healthy tissue allowing selective delivery.^[^ ^139^, ^140^ ^]^ pH‐sensitive polymers are capable of responding to alterations in surrounding environmental pH by undergoing rapid structural changes in the polymer network, including degradation, swelling, and rearrangement.^[^ ^123^ ^]^ These polymers could be utilized as reservoirs in which the drugs are released upon disassembly/deswelling associated with pH changes. As a result, studies have focused on the development of a pH‐responsive MN for site‐specific delivery of antimicrobials.^[^ ^138^, ^141^ ^]^

**Figure 6:**
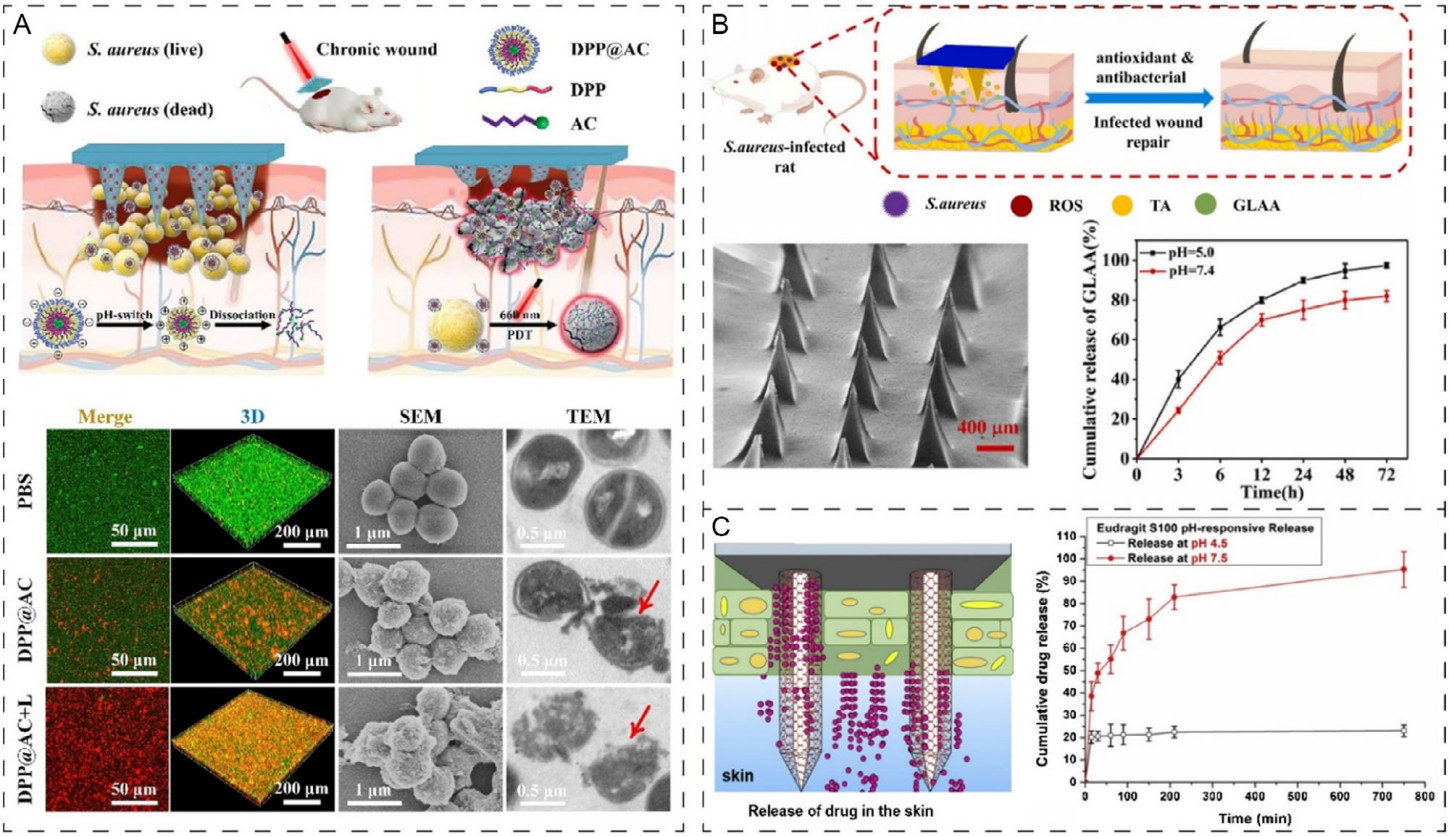
A) The MN is loaded with (DPP@AC) formed by the self‐assembly of pH‐responsive triblock polymer (DMA–PEI–PLGA, DPP) and antimicrobial peptide. The MNs can increase acidic wound pH and provide targeted release of antimicrobial peptide in response to bacteria as validated through confocal, scanning electron microscope, and transmission electron microscope. Reproduced with permission.^[^ ^142^ ^]^ Copyright 2023, Elsevier. B) MN patch comprising antioxidant backing layers loaded with antibacterial and antioxidant agents, utilizing a pH‐dependent release mechanism as part of its antibacterial strategy. This innovative approach holds significant promise for antibacterial sterilization and demonstrates excellent biocompatibility. Reproduced with permission.^[^ ^143^ ^]^ Copyright 2023, Elsevier. C) The development of PLGA‐based MNs for pH‐responsive release of the model drug in response to the wound environment showing high sensitivity to the localized wound pH condition. Reproduced with permission.^[^ ^144^ ^]^ Copyright 2021, Elsevier.

Lei and colleagues developed pH‐responsive biodegradable MNs for controlled delivery of AMPs for wound biofilm as summarized in **Figure** **6**A.^[^ ^142^ ^]^ The study used a triblock of a pH‐responsive polymer 2,3‐dimethylmaleic anhydride–polyethyleneimine–PLGA copolymer (DMA–PEI–PLGA, DPP) and the photosensitizer chlorin e6 (Ce6) linked to the Ce6‐coupled AMP loaded into a hyaluronic acid methacryloyl. The study showed that the weakly acidic environment protonated the PLGA block tertiary amine, making it hydrophobic to hydrophilic, which caused the disintegration of the micelle and the subsequent release of the drug. The in vitro and in vivo experiments showed that the MN patch applied to the *S. aureus* biofilm‐infected diabetic mice wounds and irradiated with laser showed excellent biological response in response to both bacterial clearance and wound healing progression. The AMP and photodynamic activation achieved synergistic biofilm eradication and improved wound re‐epithelialization after 15 days providing a safer and attractive pH‐responsive approach.

A similar study reports an antibiotic‐free MN patch consisting of antioxidant backing layers loaded with antibacterial and antioxidant antibacterial (Figure 6B).^[^ ^143^ ^]^ The backing layer uses CMC serving as a wound‐promoting matrix that revitalizes the impaired wound‐healing process. The application of the MN shows a pH‐dependent release mechanism and holds great promise in antibacterial sterilization. Similarly, Asad Ullah and colleagues proposed a simpler approach for pH‐responsive MN delivery. The study used a porous PLGA layer on stainless steel MNs with the loading of drugs and fluorescent dye inside the pores as shown in Figure 6C.^[^ ^144^ ^]^ The drug was encapsulated in the pores of the porous layer using aqueous gelatin porogen, and the pores containing the drug were capped by applying a thin film of Eudragit S100. The Eudragit S100 is a pH‐responsive polymer that is dissolved in contact with interstitial or wound fluid at alkaline pH releasing the payload to the affected area/wound bed. The pH‐dependent release was evident through 76% release of drugs at pH 7.5 compared to 20% in healthy simulated wounds at pH 4.5. The MN was also validated using the Sprague‐Dawley (SD) rat model indicating strong penetration and retention of model dye within the wounds. Another study has also used Eudragit S‐100 to generate dissolving pH‐responsive MNs showing high pH responsiveness and MN functionality in the context of wound infection management.^[^ ^145^ ^]^

AMPs are widely used for antibacterial applications. However, AMP biological properties are significantly influenced by the delivery method where AMP function is highly sensitive to degradation in protease rich wound microenvironment.^[^ ^146^ ^]^ A recent study has reported the development of pH‐responsive MNs to improve the delivery of AMP and recombinant type III humanized collagen (Col III).^[^ ^147^ ^]^ The study was designed to achieve slow and targeted release of AMPs to be effective against bacterial infection in the deep wound tissue. The acidic environment of an infected wound caused MN conformational change to disintegrate and promote the release of the therapeutics resulting in bacterial eradication. The study reported that AMPs were released by responding to the infected microenvironment to efficiently kill bacteria and deliver Col III, which is known for anti‐scarring benefits and promotes tissue repair and regeneration.

#### 3.3.3 Glucose‐Responsive MNs

Diabetes mellitus (DM) is a metabolic disease characterized by high blood glucose levels that increases the prevalence of several diseases including chronic wound conditions.^[^ ^148^ ^]^ Patients with DM are required to inject insulin subcutaneously by themselves several times a day to control blood glucose levels. Chronic skin wounds that fail to heal in diabetic patients represent a prevalent and serious health concern, significantly affecting patient morbidity and mortality rates.^[^ ^149^ ^]^ Diabetic patients with chronic wounds face persistent pain, discomfort, and infection which often lead to the development of necrosis and gangrene and lead to amputations or require a high degree of clinical consultations, dressings, and hospitalizations, ultimately resulting in poor quality of life with reduced life expectancy.^[^ ^150^ ^]^

Infected diabetic ulcers are highly susceptible to serious complications including bacterial infections, excessive inflammation, hypoxia, and impaired biological responses.^[^ ^151^ ^]^ As a result, the elevated glucose levels in the wound microenvironment, combined with prolonged inflammation, lead to a significant risk of severe infection, a condition prevalent in more than 90% of diabetic patients.^[^ ^7^ ^]^ The prolonged inflammation produces large amounts of ROS, with aggravative oxidative stress that detrimentally impacts wound healing and angiogenesis.^[^ ^152^ ^]^ Therefore, it is imperative to holistically manage diabetic wounds by controlling glucose levels and removing biofilms that result in the development of clinical infection. Glucose‐responsive MNs have been explored as a promising approach to not only deliver antimicrobials against biofilms but also dynamically respond based on wound glucose levels. These MN can be engineered to release therapeutic agents, growth factors, or other bioactive substances in response to increased glucose levels. These MNs can dissolve in response to glucose and release the therapeutics, enhancing targetability, tissue permeation, and efficacy however yet to be demonstrated effective in clinical trials.

**Figure 7:**
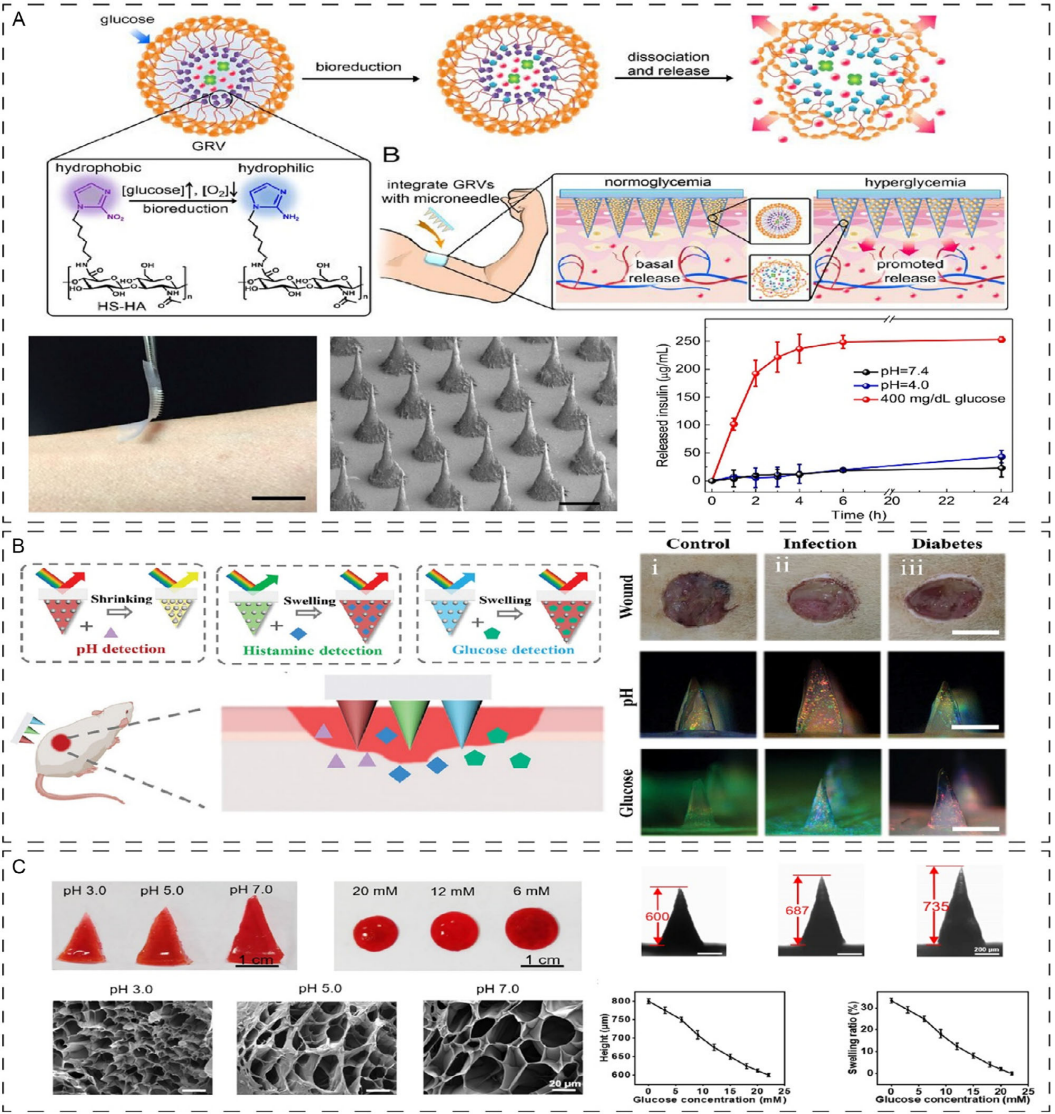
A) Glucose‐responsive insulin delivery using hyaluronic acid MN patch integrated with insulin and glucose oxidase. The study has taken advantage of the local generation of hypoxia due to the consumption of oxygen in the enzymatic reaction as a trigger for rapid insulin release in response to hyperglycemia. Reproduced with permission.^[^ ^153^ ^]^ Copyright 2015, National Academy of Sciences, B) A glucose‐sensitive MN screening for wound biomarkers including pH and glucose providing accurate qualitative measurements. Reproduced with permission.^[^ ^158^ ^]^ Copyright 2023, Wiley. C) A unique study demonstrates that MNs can penetrate deep tissue while providing a fast response to glucose levels in the physiological range. The in situ extraction of glucose from ISF provides minimally invasive information on glucose concentration by adjusting the MN's height and swelling as indicators of glucose concentration. Reproduced with permission.^[^ ^159^ ^]^ Copyright 2023, Elsevier.

A study by Yu et al. reports a glucose‐responsive insulin delivery using a hyaluronic acid MN patch integrated with insulin and glucose oxidase as summarized in **Figure** **7**A.^[^ ^153^ ^]^ The study has taken advantage of the local generation of hypoxia due to the consumption of oxygen in the enzymatic reaction as a trigger for rapid insulin release in response to hyperglycemia. In response to these microenvironments, the vesicle dissociates and releases encapsulated insulin under the local hypoxic environment, caused by the enzymatic oxidation of glucose in the hyperglycemic state to regulate glucose and alleviate the challenges of nonhealing chronic wounds including inflammation and hypoxic environment. Another study proposes a simpler approach without the use of any external antimicrobial agents.^[^ ^154^ ^]^ The glucose‐responsive MNs composed of GelMa, glucose‐responsive monomer 4‐(2‐acrylamidoethylcarbamoyl)‐3‐fluorophenylboronic acid and gluconic insulin (G‐insulin). The glucose‐responsive hydrogel MNs showed high biocompatibility, glucose‐responsive insulin release, and desirable characteristics for strong wound adhesion serving as a multimodal wound dressing. The application of the MNs accelerated the diabetic wound healing process and improved blood glucose levels compared to controls with favorable outcomes in both eradication of bacterial infection and tissue repair. Similarly, another study has reported glucose‐responsive MNs for effective delivery of metformin to lower glucose levels.^[^ ^155^ ^]^ On‐demand delivery of insulin triggered by the changes in glucose levels has been achieved through the use of glucose transporter molecules, thereby efficiently regulating blood glucose levels.^[^ ^156^ ^]^ Metal‐organic framework (MOF)‐based glucose‐responsive MNs are also used to consume the excess glucose from diabetic wounds. The glucose oxidase was used to consume glucose in infected diabetic wounds to yield gluconic acid and hydrogen peroxide which further generated OH as a potent antibacterial agent in diabetic wound healing.^[^ ^157^ ^]^

MNs have also been used for diagnosing and accurately monitoring glucose levels. A recent study reported using MNs as multiple screening of wound small molecules including pH, glucose, and histamine, all playing critical roles in wound healing (Figure 7B).^[^ ^158^ ^]^ The glucose sensing was achieved using fluorophenylboronic acid (FPBA), a glucose‐responsive moiety. The MN system would convert physiological molecule levels into structural color signals providing qualitative measurements to detect target molecules. A similar study reported dual‐responsive pH and glucose‐responsive hydrogel MNs (Figure 7C).^[^ ^159^ ^]^ The hydrogel MN composed of GelMA together with pH‐sensitive moiety and glucose oxidase showed that MNs can penetrate sufficiently and provide a fast response to glucose levels in the physiological range. The in situ extraction of glucose from ISF provides minimally invasive information on glucose concentration by adjusting the MN's height and swelling. The MN technology was validated using a diabetic mice model.

In the context of clinical applications, glucose‐responsive MNs are emerging as invaluable tools, extending beyond their conventional role in insulin delivery. This evolving paradigm extends to the diagnostic arena, where MNs may prove not only to be effective at delivering insulin but also in detecting early indicators and biomarkers of diabetic ulcer development, thereby bolstering diagnostic capabilities. Current advancements in MN demonstrate a highly important dual functionality, smoothly combining diagnostic and therapeutic features for addressing complex diabetic wound conditions and developing theragnostic tools for wound management applications. These approaches often feature insulin‐responsive elements, ensuring optimized release based on specific requirements, therefore preventing excessive glucose accumulation or dysregulation. The current emphasis lies in crafting optimized MN systems, characterized by controlled and sustained insulin release with an extended duration of action underscoring the potential of glucose‐responsive MN systems in transformative clinical settings for the effective and safe treatment of diabetic wounds including infection associated with diabetic foot ulcers.

#### 3.3.4 ROS‐Responsive MNs

**Figure 8:**
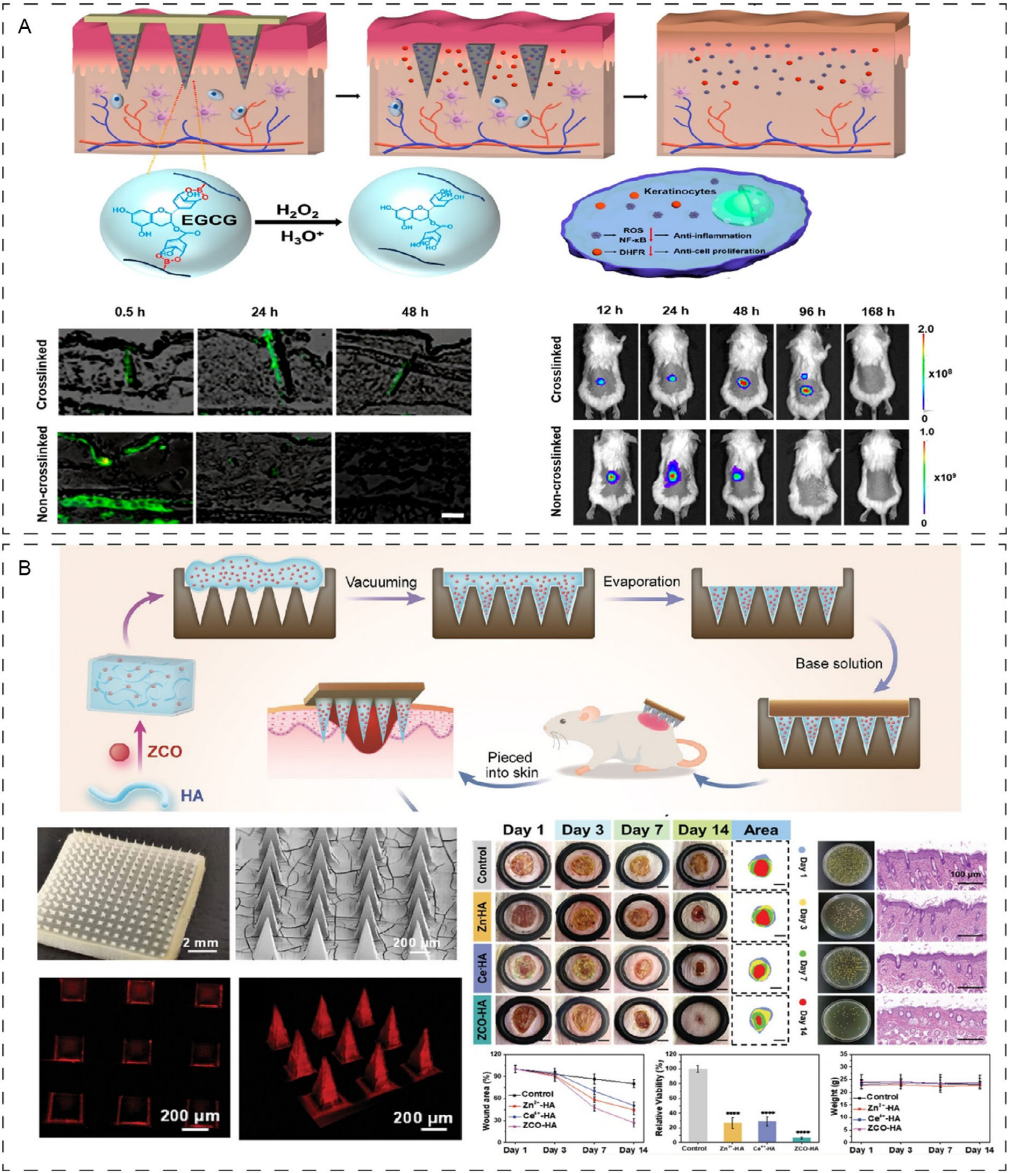
A) The development of a ROS‐responsive detachable MN patch loaded with anti‐inflammatory drugs in a cross‐linked polymer needle tip for the treatment of chronic inflammation in psoriasis while being validated using in vitro and in vivo models. Reproduced with permission.^[^ ^165^ ^]^ Copyright 2023, American Chemical Society. B) development of a multicomponent enzyme‐responsive natural polymer‐based hyaluronic acid MNs loaded with cerium/zinc‐based nanomaterial (ZCO) for the treatment of diabetic wounds through controlling oxidative stress to alleviate wound inflammation and accelerate diabetic wound closure after 14 days of ZCO‐MN application. Reproduced with permission.^[^ ^166^ ^]^ Copyright 2023, WIley.

In chronic wounds, prolonged inflammation and impaired tissue repair mechanisms can lead to persistent oxidative stress,^[^ ^160^ ^]^ which studies to date have established that elevated levels of ROS are detrimental causing damage to cellular components, including proteins, lipids, and DNA.^[^ ^161^ ^]^ ROS plays a key role by augmenting inflammation and can result in excessive cellular apoptosis. Elevated and prolonged oxidative stress and reduced antioxidant capacity in infected wounds can lead to redox imbalance, thereby delaying healing.^[^ ^162^ ^]^ Hence, ROS levels play a vital role in cellular proliferation, migration, and adhesion, which are vital mechanisms for normal wound healing.^[^ ^163^ ^]^ Researchers have looked at different ways to explore ROS modulation and often clearance is necessary to ensure the effectiveness of healing.^[^ ^164^ ^]^ Therefore, developing a ROS‐responsive MN delivery system to alleviate oxidative stress and relieve the inflammatory environments has provided opportunities to promote the refractory wound healing process. A recent study conducted by Duohang et al. developed a H_2_O_2_‐responsive detachable MN patch with MTX and epigallocatechin‐3‐gallate loaded in cross‐linked polymer gel needle tips for the treatment of chronic inflammation in psoriasis (**Figure** **8**A).^[^ ^165^ ^]^ The increased levels of ROS and acidity within the wound environment prompted a sustained release of therapeutics, effectively mitigating inflammation. The in vivo application showed that the ROS‐responsive MN efficiently delivered antiproliferative and anti‐inflammatory drugs resulting in improved treatment outcomes after 7 days of application against a psoriasis murine model. The dysregulation of ROS is associated with uncontrolled oxidative stress, which plays a pivotal role in sustaining and deregulating the pathogenesis of chronic wounds. Hence, MNs are also known to play a vital role in regulating oxidative stress. Yang and colleagues developed a multicomponent enzyme‐responsive natural polymer‐based hyaluronic acid MNs loaded with cerium/zinc‐based nanomaterial (ZCO) for the treatment of diabetic wounds through controlling oxidative stress (Figure 8B).^[^ ^166^ ^]^ The multicomponent MN system can destroy the oxidation balance of bacteria, kill bacteria, and scavenge ROS to alleviate oxidative stress. The system is further supported by modulating the anti‐inflammatory response mechanism to promote cell proliferation, migration, and angiogenesis, resulting in accelerated diabetic wound healing.

Along with a similar approach to modulating oxidative stress, Yu et al. reported a multi‐enzyme MN system that combines enzymatic biofilm disruption with glucose oxidase‐based chemodynamic therapy.^[^ ^167^ ^]^ The study reports that glucose oxidase converts wound glucose to H_2_O_2_ subsequently leading to decreased inflammation. The system is also integrated with α‐amylase which disintegrates the EPS structure, making bacteria vulnerable to the treatment. Therefore, this system structurally disrupts biofilms, consumes excess glucose to self‐supply H_2_O_2_, and reduces bacterial infections. MNs are gaining attention for different transdermal applications targeting key pathological changes of oxidative stress. Zhang and colleagues used ROS‐responsive MNs for an anti‐acne treatment.^[^ ^168^ ^]^ A PVA was cross‐linked using a dual phenylboronic acid contained linker, which is cleaved by ROS through oxidation and hydrolysis. The MN system was able to deliver antibiotics triggered by the ROS generated from *Propionibacterium acne* bacteria, inhibited their growth, and improved acne treatment. ROS‐responsive MNs are also reported for pulmonary fibrosis.^[^ ^169^ ^]^ ROS‐triggered drug release was achieved by coupling a biocompatible PVA linked with a ROS‐responsive molecule. The molecule is oxidized and hydrolyzed when exposed to elevated levels of ROS during pathological conditions releasing the epithelial‐specific integrin that is a receptor for the extracellular matrix (integrin αvβ6)‐blocking antibody to treat pulmonary fibrosis. The study reports that the hydrogen‐peroxide‐responsive MN is biodegradable for smart delivery of integrin‐αvβ6‐blocking antibody offering a rapid response, excellent biocompatibility, protection of bioactivity, high tissue permeation, and specific targeting to lesions, features that would be desirable in MN design for many conditions including infection control of chronic wounds.

#### 3.3.5 Applications of MNs for the Management of Chronic Infected Wounds

The increasing demand for personalized therapeutics has led to the development of diverse MN types catered to specific requirements including for the management of infected chronic wounds. MNs can be tailored based on the wound condition requirements at different steps of wound management including treatment with controlled and instant drug delivery using small, and large, NPs, proteins, peptides, and cells.^[^ ^170^ ^]^ MNs are often developed tailored to the wound condition providing a conducive environment for tissue regeneration. For example, two‐layered MNs are proposed, where the tip and backing layers serve vital functions, not only delivering drugs but also providing wound‐healing properties.^[^ ^23^ ^]^ Given the complexity and interconnected biological processes that drive wound healing much more needs to be done to address the major gaps in tissue repair. Currently, most of the MNs developed for wound management applications are explored as drug delivery systems. As reflected in the past decades, MNs have been used in research preclinical studies for direct delivery of antimicrobials to disrupt biofilms, alleviate wound inflammation, increase oxygen for angiogenesis, improve cell proliferation, and modulate immune response all significantly benefiting the infection control in wound management. Hence, MNs are continually transforming the landscape of wound treatment, extending the potential for broader applications in dermatology including anti‐hemostatic,^[^ ^171^ ^]^ anti‐scaring,^[^ ^172^ ^]^ diabetic wounds,^[^ ^173^ ^]^ and addressing both acute and chronic biofilm‐related wounds.^[^ ^174^ ^]^

MNs also contribute significantly to personalized wound diagnosis, providing real‐time insights into the wound microenvironment.^[^ ^175^ ^]^ MNs can be integrated with sensors and devices to monitor the physiological parameters in real time, providing accurate and reliable information about wound healing to assist in diagnosis and treatment adjustment during wound management.^[^ ^176^ ^]^ The point‐of‐care (POC) testing offers rapid and accurate results allowing decentralized diagnosis and personalized medicine. This timely and reliable information vitally supports clinicians with decision‐making for better management of infection. Traditional diagnosis of infection is subjective and microbiology testing is time‐consuming. Additionally, the noninvasive application of MNs ensures minimal discomfort, fostering patient compliance, and tailored therapy. As a result, the development of robust, rapid, and cost‐effective POC diagnostic MNs to supplement standard traditional diagnostic tools in wound management has been gaining research focus in the last decade. Validation of developed approaches in randomized clinical trials holds promise for clinical translation and applications in wound management.

### 3.4 The Significance of MNs in Wound Diagnosis

Traditional diagnostic methods for the detection of pathogens are time‐consuming processes, rely on centralized laboratories, need experienced personnel, and expensive equipment, and are also often expensive and inaccessible to general populations.^[^ ^177^ ^]^ For example, biofluids including blood and plasma have been long known as the gold standard for definitive diagnosis, which often requires the use of invasive finger pricking and hypodermic needle extraction causing pain, bruising, and possible infection. This has sparked interest in using POC MN diagnostics for minimally invasive wound infection diagnosis and real‐time monitoring in clinical settings. Currently, wearable hydrogel dressings are being developed to detect and monitor bacterial infections providing continuous data on important wound biomarkers including pH,^[^ ^178^ ^]^ temperature,^[^ ^179^ ^]^ and ROS,^[^ ^180^ ^]^ all indicative of bacterial infection. This could be a significant step toward better wound diagnosis and treatment. While imaging tools like the MolecuLight i:X (wound imaging device) are also increasingly recognized for bacterial infection detection they are limited to only detecting superficial infections.^[^ ^181^ ^]^ Hence, there is a growing potential for MNs to offer innovative approaches to enhance drug delivery and wound diagnosis. This technology enables efficient penetration of tissues at desired depths, accessing blood, ISF, or wound fluid. This promises accurate infection diagnosis and targeted treatment of biofilms within deep wound tissue using POC technologies. The POC device technologies are expected to have significant market potential as the clinical field transitions toward more advanced, intelligent, accurate, and readily accessible diagnostic technologies. For example, POC MN has been useful for hematological analysis of real‐time blood diagnosis.^[^ ^182^ ^]^ In contrast, ISF or wound fluid contains a wide range of important physiological biomarkers including ions, glucose proteins, and cells, and can be used to monitor changes in the wound microenvironment. Hence, employing MN for accessing wound biomarkers shows great promise in enhancing health monitoring, assisting clinicians in precisely diagnosing patients at an earlier stage of infection development or pathogen colonization. Most importantly, this approach has been shown to determine the concentration of blood glucose levels, significantly benefiting diabetic patients.^[^ ^183^ ^]^ Smart MNs offer a promising way to speed up diagnosis, providing rapid medical information for faster decision‐making in patient care. The wearable theragnostic MN technology holds promise for personalized healthcare in future medicine, but several challenges must be addressed before its widespread clinical use in managing chronic wounds and beyond.

## 4 Challenges and Opportunities in Clinical Translation

The global market for wound care products continues to skyrocket expected to be 27.8 billion USD by 2026 with increased emphasis on patient acceptability, and implementation into clinical practice.^[^ ^184^ ^]^ Hence, the concept of using MN technology in healthcare continues to gain medical attention opening exciting avenues for clinical translations. Despite the significant research efforts, the translation process continues to face challenges ensuring that MNs meet regulatory standards and can be seamlessly integrated into medical practice and standards of care. To determine the progress of MNs in clinical trials, a comprehensive literature search was conducted across multiple databases, including, ClinicalTrials.gov, the EU Clinical Trials Register, and Australian New Zealand Clinical Trials Registry. The data acquired from these databases has been tabulated in **Table** **2** collectively summarizing the status of major MN under clinical development and progression toward clinical translation for wound‐related applications in the last 5 years.

**Table 2:** Summary of completed and ongoing clinical trials of MNs for transdermal delivery targeting wound conditions in the last 5 years.

Condition	Therapeutics used for delivery	Types of MNs	Clinical trial no.	Phase	Status
Healthy volunteers	Penicillin delivery	Metallic MN	NCT04053140	Phase 1	Recruiting
Recessive dystrophic epidermolysis bullosa (RDEB)	Gentamicin	SolidMR2 microneedle roller device	NCT03012191	Phase 2	Completed
Postsurgical scars	Small interfering RNA (SiRNA)	Dissolvable	NCT06138964	Phase 3	Recruiting
Skin inflammation——contact dermatitis	Squaric acid dibutyl ester (SADBE) + dupilumab	Dissolvable microneedle	NCT05535738	Phase 3	Recruiting
Chronic plaque psoriasis vulgaris	Skin biopsy collection	Coated MN	NCT03795402	N/A	Completed
Glucose measurements (diabetes)	Glucose	Hydrogel MNs	NCT02682056	N/A	Completed

Interestingly, based on these findings, there are currently no clinical trials underway to test MNs specifically for treating wound infections. However, several trials are exploring the use of MNs to deliver antimicrobials and therapeutics for managing hereditary chronic and acute inflammatory skin conditions, including epidermolysis bullosa (EB) and psoriasis. The lack of clinical translation for the management of infected wounds is believed to be partly due to a limited understanding of MN safety, quality standards, and good manufacturing practices. Additionally, the industrialization of MN technology faces additional barriers due to the high production costs and the absence of appropriate models to consistently validate permeability, safety, and efficacy.^[^ ^185^ ^]^ Additionally, the appropriate design of randomized clinical trials to validate MN safety and efficacy is required for clinical translation rather than single case studies. However, the prospects of MNs for cosmetics, monitoring, and diagnostics are promising. The majority of the MN technology that has progressed for clinical use is for cosmetic products including Derma roller, MicroHyala, and Dermapen. These technologies are fabricated using solid MN except MicroHyala, which is a dissolving application.^[^ ^186^, ^187^ ^]^ They all remain popular in the cosmetic industry used for anti‐wrinkle, anti‐scarring, fine lines, and loose skin. For example, derma roller has been widely used for transdermal delivery of many drugs (ibuprofen, diclofenac, etc.) or for skin pretreatment for cosmetic procedures to enhance formulation permeation and drug bioavailability.^[^ ^188^, ^189^ ^]^ Additionally, other marketed and commonly available MN delivery systems are also summarized in a recent review highlighting MN technology impact across different fields.^[^ ^190^ ^]^

Current commercially available MN sensors have been employed for insulin delivery and glucose monitoring, with notable examples including Dexcom G6, FreeStyle Libre, and Medtronic MiniMed 670G.^[^ ^191^, ^192^ ^]^ These advanced technologies have revolutionized glucose monitoring by eliminating the need for traditional finger‐prick blood sampling. By continuously monitoring glucose levels through the subcutaneous implantation of a needle‐type biosensor, these devices provide continuous and noninvasive glucose monitoring. In addition, MicroJet600 and Macroflux have also been popular for intradermal immunizations, protein, and anesthetic delivery.^[^ ^193^ ^]^ However, there are a lot of MNs under different phases of clinical trials for vaccination and diabetes management, ISF measurements, local anesthetics, acne, and skin conditions, awaiting full‐scale industrialization, and commercialization.^[^ ^194^ ^]^

**Figure 9:**
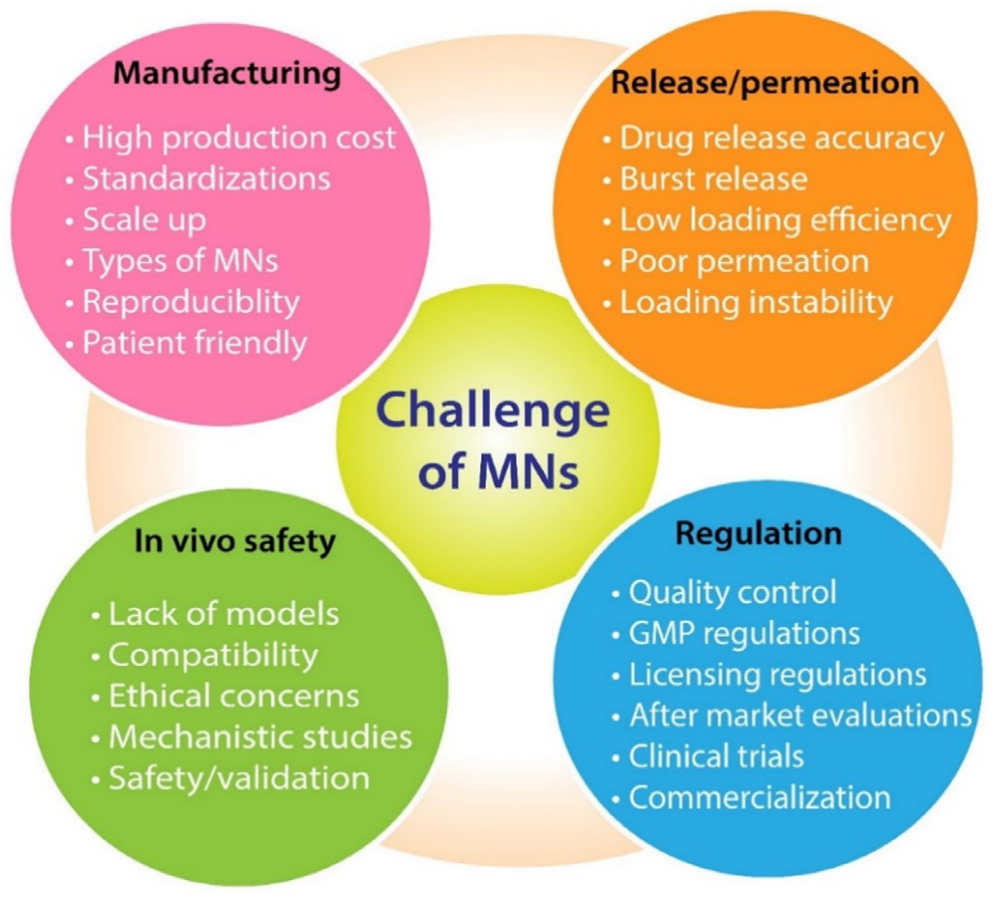
Summary of current challenges of MNs in pursuit of clinical translation.

For the last two decades, MNs have continuously progressed and diversified in different fields whether for therapeutics, diagnostics, or sampling. Despite this positive progress, compared to hydrogel applications, MN technology is still relatively new and hence faces many challenges and hurdles in widespread clinical implementation. The major challenges revolving around the development and application of MNs are summarized in **Figure** **9**. To promote the commercialization of MNs, it is important to rectify and address major current issues of MN devices for wide‐scale implementation concerning technology safety, patient compliance, production, and market potential.^[^ ^189^ ^]^ Current issues arise from the selection of suitable fabricating materials to provide sufficient mechanical strength with high loading efficiency for sustained and long‐term applications ensuring accurate and sufficient therapeutic drug index within affected sites. In addition, MN technology continues to face challenges in practical implementation, including the depth of skin/wound bed penetration, patient compliance while being user‐friendly and having long‐acting activity all of which are paramount for severe wound‐related infections, while maintaining industrial requirements of high and complex manufacturing with scalable production and long shelf life.^[^ ^195^ ^]^ This also relates to MN's ability to deliver a variety of drug molecules including hydrophilic, hydrophobic, and high molecular weight, NPs with maximum permeability, stability, and bioavailability through different layers of the skin/wound bed. A recent review summarizes the prospects and challenges of MNs for various skin conditions.^[^ ^92^ ^]^

Additionally, the application of MNs needs to be safe for wound insertion without any breakage of the materials.^[^ ^196^ ^]^ For example, the application of hollow MNs could raise some technical issues of needle blockage, drug leakage, or needle clogging thereby reducing drug delivery and dosage accuracy. Another area of concern is that MNs may create microchannels that could potentially lead to increased incidence of bacterial reinfection. However, some have argued that the microchannels are healed at a significantly faster rate without the risk of infection compared to traditional hypodermic needles.^[^ ^197^ ^]^ The risk of infection is also reduced by disinfecting the application site before and after applications and avoiding the reuse of the application device. Additionally, developing user‐friendly, self‐administered MN devices could empower patients to manage treatment regimens at home, hence having the potential to significantly reduce the burden on the healthcare systems.

In addition to simple operation, and accessibility, the safety for human applications is also an important aspect of the future widespread application of MNs and clinical implementation in wound management. Nguyen and colleagues have recently summarized the application, safety profile, regulatory issues, and the future of MNs using biopharmaceutical agents.^[^ ^198^ ^]^ The use of safe and biocompatible materials would be ideal to accelerate the development speed and efficiency. In general, it is widely accepted that MN applications are well tolerated only known to create transient, reversible, superficial, and localized microinjuries. In addition, addressing sterilization methods to ensure aseptic application and overcoming challenges related to scale‐up and manufacturing are other crucial steps toward mitigating these limitations and fostering broader clinical integration. Therefore, improvements in the manufacturing processes are critical for expanding production capacity and bolstering process control to ensure product quality while propelling advancements in therapeutic interventions and diagnostics. Knowing the stringent regulatory requirements, it is important to collectively standardize the protocols related to the production and validation of the MNs with well‐supported randomized clinical trials, paving the way for their industrialization, clinical implementation, and accessibility across diverse global healthcare facilities. Table 2 presents a summary of completed and ongoing clinical trials of MN for transdermal delivery in different wounds, while **Table** **3** provides an overview of the latest MN designs and applications in wound management discussing the advantages and disadvantages of each approach.

**Table 3:** Summary of latest antimicrobial microneedles design and application for wound management.

Drug or therapeutic	Types of MNs	Wound models	Advantages	Disadvantages	References
CRRI3 antimicrobial peptide (AMP) and manganese oxide nanoparticles	GelMA microneedles	*Staphylococcus aureus* infected full‐thickness skin defect model in rats (15 day model)	Double‐layered MN design Dual wound activity Stimuli‐responsive activity	High‐cost and complex synthesis Not dissolvable material	[99]
AMP (W379) and monoclonal antibodies	PVP microneedles	Diabetic mouse wound *S. aureus* biofilm model (5 day model)	Dual antibacterial mechanism Good loading capacity	Limited wound healing effect Short‐term analysis	[100]
Calcium peroxide and AgNPs	Silk fibroin methacryloyl hydrogel microneedle	Type 1 diabetic wound mouse model (12 day model)	Multifunctional properties Sustained release Applicable to other delivery systems Anti‐inflammatory effects	May suffer poor mechanical strength Low loading capacity may limit clinical applications	[102]
Luteolin and nitric oxide (NO) donor L‐arginine (L‐Arg)	Sodium hyaluronate (HA)	*S. aureus* SD wound rat model (8 day model)	Biofilm permeability and dispersion Applicable to all types of wounds	Complex preparation High source of photothermal application may lead to burn injury/erythema	[104]
AMP W379	PVP and PCL microneedles	Type II diabetic mouse wound infection model (3 day model)	Biodegradable Biocompatible materials High penetration The potential for scale‐ups	High cost of AMPs Potential for low AMP stability in protease‐rich wound environment Low number of mice used	[76]
Calcium peroxide	PVP and PET substrate	Ex vivo porcine skin biofilm model (36 h model) on polymicrobial infections.	Flexible substrate Anti‐biofilm effect Wound oxygenation	Lack of in vivo data Limited wound healing effects unclear effect on inflammation	[105]
Lipoic‐acid sodium‐protected selenium NPs (basal), Fe_3_O_4_ in the tip	Methacrylate hyaluronic acid	Diabetic mouse *S. aureus* wound infection model (14 day model)	Deep biofilm penetration Alleviate wound conditions Dual‐layered MN	Electromagnetic utility High cost and operations Degree of user variability	[107]
AgNPs, glucose oxidase nanocapsules, apramycin	PVA	*Propionibacterium acne*‐induced inflammation mice model (scarless skin model) (6 day model)	Triple component therapy Synergistic antibacterial activity	Too complex and inconsistent The use of antibiotics Further research is required to validate therapeutic efficacy	[117]
Fe_3_O_4_ nanoparticles on graphene oxide nanosheets	Methacrylated hyaluronic acid, photothermal MN	Diabetic mouse wound model (15 day model)	High mechanical strength High antibiofilm properties Wound regulations	Complex fabrication High heat‐induced treatment Not all chronic wounds require iron modulation therapy	[130]
Human epidermal growth factor (hEGF)	Silk fibroin, polyurethane (shark teeth inspired MN)	Diabetic mouse's foot model (9 day model)	Cost‐effective synthesis Good loading efficiency	Limited antibacterial effect Not applicable to flat wounds Complex synthesis	[131]
AMP‐Ce6, AC	Hyaluronic acid methacryloyl hydrogel MN‐pH responsive	*S. aureus‐*biofilm‐infected diabetic mice (15 day model)	Potential for complete wound therapy Good pH‐responsive release Precise bacterial targets	Fast MN dissolution limiting long‐term efficacy	[142]
Fluorophenylboronic acid (FPBA) Polyacrylamide (PAM)	polyethylene glycol diacrylamide (PEGDA)	Wound SD rat model infected with *S. aureus* (24 h model)	Multiple biomarker detection Potential for real‐time monitoring	Limited for only diagnostic purposes Semi‐qualitative data	[158]
Cerium/zinc‐based nanomaterial (ZCO)	Hyaluronic acid (HA)	Diabetes wound healing model (14 day model)	Suitable for all types of chronic wounds Ability to regulate wound environment. Good mechanical strength	Lack of data for biofilm eradication efficacy Long‐term application may cause toxic effects	[166]
*Lactobacillus reuteri* (*L. reuteri*) probiotic	Polyvinyl alcohol (PVA), sucrose, 5% glycerol	Full‐thickness skin mouse wound model (9 day model)	High strength Long‐acting application	Lack of immunogenicity data	[205]
Cerium dioxide (CeO_2_) Mesoporous silica NP Silver ions	γ‐PGA	Diabetes wound healing model (12 day model)	Targeting multiple parameters to regulate wounds High strength and penetration Wound healing and anti‐inflammatory Potential for deep tissue penetration	The complex process of preparation Therapeutic release is limited to diabetic wound conditions	[206]

## 5 Conclusion and Perspectives

In this review, we have summarized the current progress, design strategies, prospects, and applications of MNs for transdermal antimicrobial drug delivery. MN delivery system with optimized characteristics for wound application is currently becoming one of the hot spots in the field of drug delivery to fight against chronic infections and associated clinical challenges. This is because antibacterial resistance infection continues to pose a substantial global health threat with a pressing need for the exploration and development of alternative strategies. This imperative arises from the limitations and challenges associated with the conventional approaches to the management of wound infection. Over the years, the quest for innovative solutions has become important in addressing the escalating threat of antibacterial resistance, ensuring that the formulation is effective and sustainable for infection control. As the landscape of treatment approaches changes, there is always a need for more accurate, precise, and personalized strategies as a sustainable healthcare platform.

For the last two decades, MNs have been the subject of significant research for the delivery of vaccines, biologics, cosmetics, and other small molecules for diagnosis, and treatment of diseases. However, there are no MNs approved for wound healing applications, with current primary clinical use focused on cosmetics, vaccines, and other industries. The increased activity and commercialization in those sectors serve as evidence of MN technology appeal to both the healthcare industry and patients. Early clinical trials validated the potential of MNs to enhance drug absorption through the skin, leading to increased interest and investment in this technology across various fields, with ongoing research efforts. Despite the lack of clinical approvals for MNs in treating chronic wounds, there is growing research interest, with several potential candidates currently undergoing preclinical studies as summarized in Table 3. The current development of MNs has significantly focused on addressing major challenges of wound infection by investigating efficacy across different aspects of wound management including wound sterilization, healing, immune functions, and tissue regeneration with the potential of next‐generation MNs to serve as a complete wound care package. Moreover, there are growing preclinical and clinical trials focused on antibiotic delivery using MN technology for patients with the genetic skin blistering condition EB, who face daily challenges with wound infection. Clinical trials, including Phase 3 studies employing dissolvable MN technologies, offer hope for faster clinical translation of MN technology for infection control during chronic wound management within the next decade. These trials are crucial for bridging existing clinical gaps and advancing MN technology for wound management, drug delivery, and infection diagnosis. Successful trials in this area could lead to practical applications of MNs clinically, offering tangible benefits to patients with various chronic wounds, including burn injuries, where traditional delivery methods are often unsatisfactory.

Moreover, current research emphasizes the need for integrated, closed‐loop systems that can both diagnose and treat conditions. Such theragnostic platforms are essential for accurate diagnosis and timely treatment, yet they present significant technical and regulatory challenges. This approach would not only enable the real‐time and continuous monitoring of physiological situations but also demand a smart theragnostic system that can rapidly process, wirelessly transmit, and intelligently respond to electrochemical signals remotely with high convenience. The progress in this area is exciting and would be rewarding to generate next‐generation systems incorporating the latest MN designs. Hence, MNs have a significant potential to change the landscape of clinical wound management with great emphasis on new designs and features to provide better and smarter patient support and clinical wound care management.

To date, various types of MNs have been proposed and fabricated. Depending on the intended applications, each of the MNs has advantages and characteristics tailored to specific applications and release mechanisms, drug loading, degradation, production, and safety. As highlighted, there is a significant drive toward POC MNs that can provide a smart release mechanism while being biodegradable and self‐administrable to avoid frequent application and removal. Despite the interest, most of the currently approved MNs in the cosmetic industry lack these important features. Most of the approved applications of MNs are focused on supporting vital stages of disease therapy with only a few used for diagnosis, or sampling. In the context of chronic wounds, POC MNs could benefit patients by identifying the early risk of infection through the changes in the wound microenvironment and becoming activated to provide on‐demand therapy, eliminating the risk of developing severe chronic conditions.

Even though MNs are a minimally invasive technique and safe, there are a lot of safety concerns that have been raised hindering its potential progression beyond the clinical trials for widespread patient use. The major issues surrounding MN production are summarized in (Figure 9). However, studies are heavily focused on identifying major issues to improve production, safety, delivery, and importantly patient acceptance. Several factors would determine the feasibility of an MN product entering the commercial market, including drug stability, long‐term safety, dose restriction, efficient drug delivery, Good Manufacturing Practices compliance, manufacturability, and scaling‐up process. There is also a significant emphasis placed on the need to investigate the effect of the MN material breakdown and long‐term adverse effects and safety aspects. Moreover, researchers are faced with the challenge of developing human‐skin‐tailored MNs designs while being able to simultaneously simulate MN stability, needle penetration, drug diffusion, and dissolution through reliable modeling. This will make the regulatory approvals and United States Food and Drug Administration (FDA) submissions more favorable. This effort calls on collaboration between academia, industry, inventors, patients, and regulators to create an innovative system with high translation potential, and patient compliance for future drug delivery. Therefore, it is anticipated that the use of the MN system will lead to a paradigm shift in the field of drug delivery, especially for those therapeutic agents that have previously been inaccessible to traditional techniques. As anticipated, MN technology will enable personalized medicines that improve patients’ quality of life and medications’ therapeutic effects, bringing a revolutionary change to wound management and patient care.

## Conflict of Interest

The authors declare no conflict of interest.

## References

[1] M. Falcone , B. De Angelis , F. Pea , A. Scalise , S. Stefani , R. Tasinato , O. Zanetti , L. Dalla Paola , The Lancet 2022, 26, 140. 10.1016/j.jgar.2021.05.010 34144200

[2] A. Clinton , T. Carter , Lab. Med. 2015, 46, 277. 26489671 10.1309/LMBNSWKUI4JPN7SO

[3] C. J. L. Murray , K. S. Ikuta , F. Sharara , L. Swetschinski , G. Robles Aguilar , A. Gray , C. Han , C. Bisignano , P. Rao , E. Wool , S. C. Johnson , A. J. Browne , M. G. Chipeta , F. Fell , S. Hackett , G. Haines-Woodhouse , B. H. Kashef Hamadani , E. A. P. Kumaran , B. McManigal , S. Achalapong , R. Agarwal , S. Akech , S. Albertson , J. Amuasi , J. Andrews , A. Aravkin , E. Ashley , F.-X. Babin , F. Bailey , S. Baker , et al. Lancet 2022, 399, 629.35065702

[4] M. Xie , M. Gao , Y. Yun , M. Malmsten , V. M. Rotello , R. Zboril , O. Akhavan , A. Kraskouski , J. Amalraj , X. Cai , J. Lu , H. Zheng , R. Li , Angew. Chem., Int. Ed. 2023, 62, 202217345. 10.1002/anie.202217345 36718001

[5] U. Theuretzbacher , K. Outterson , A. Engel , A. Karlén , Nat. Rev. Microbiol. 2020, 18, 275. 31745331 10.1038/s41579-019-0288-0 PMC7223541

[6] G. Han , R. Ceilley , Adv. Ther. 2017, 34, 599. 28108895 10.1007/s12325-017-0478-y PMC5350204

[7] K. Rahim , S. Saleha , X. Zhu , L. Huo , A. Basit , O. L. Franco , Microb. Ecol. 2017, 73, 710. 27742997 10.1007/s00248-016-0867-9

[8] K. Razdan , J. Garcia‐Lara , V. R. Sinha , K.K. Singh , Drug Discovery Today 2022, 27, 2137. 35489675 10.1016/j.drudis.2022.04.020

[9] O. Ciofu , C. Moser , P Ø Jensen , N. Høiby , Nat. Rev. Microbiol. 2022, 20, 621. 35115704 10.1038/s41579-022-00682-4

[10] D. Sharma , L. Misba , A. U. Khan , Antimicrob. Resist. Infect. Control 2019, 8, 76. 31131107 10.1186/s13756-019-0533-3 PMC6524306

[11] H. Haidari , R. Bright , Z. Kopecki , P. S. Zilm , S. Garg , A. J. Cowin , K. Vasilev , N. Goswami , ACS Appl. Mater. Interfaces 2022, 14, 390. 34935355 10.1021/acsami.1c21657

[12] J. M. V. Makabenta , A. Nabawy , C.‐H. Li , S. Schmidt‐Malan , R. Patel , V. M. Rotello , Nat. Rev. Microbiol. 2021, 19, 23. 32814862 10.1038/s41579-020-0420-1 PMC8559572

[13] X. Ding , Q. Tang , Z. Xu , Y. Xu , H. Zhang , D. Zheng , S. Wang , Q. Tan , J. Maitz , P. K. Maitz , S. Yin , Y. Wang , J. Chen , Burns Trauma 2022, 10, tkac014. 35611318 10.1093/burnst/tkac014 PMC9123597

[14] V. Choi , J. L. Rohn , P. Stoodley , D. Carugo , E. Stride , Nat. Rev. Microbiol. 2023, 21, 555. 37258686 10.1038/s41579-023-00905-2

[15] D. G. Metcalf , D. Parsons , P. G. Bowler , Int. Wound J. 2017, 14, 203. 27004423 10.1111/iwj.12590 PMC7949869

[16] H. Haidari , S. Garg , K. Vasilev , Z. Kopecki , A. Cowin , Wound Pract. Res. 2020, 28, 176.

[17] G. Yaşayan , O. Nejati , A. F. Ceylan , Ç Karasu , P. Kelicen Ugur , A. Bal‐Öztürk , A. Zarepour , A. Zarrabi , E. Mostafavi , Appl. Mater. Today 2023, 32, 101829.

[18] P. J. Buch , Y. Chai , E. D. Goluch , Clin. Microbiol. Rev. 2019, 32, 10.1128/cmr.00091-18. PMC6431129 30651226

[19] D. A. Williamson , G. P. Carter , B. P. Howden , Clin. Microbiol. Rev. 2017, 30, 827. 28592405 10.1128/CMR.00112-16 PMC5475228

[20] T. N. Demidova‐Rice , M. R. Hamblin , I. M. Herman , Adv. Skin Wound Care 2012, 25, 304. 22713781 10.1097/01.ASW.0000416006.55218.d0 PMC3428147

[21] T. Cui , S. Wu , Y. Sun , J. Ren , X. Qu , Nano Lett. 2020, 20, 7350. 32856923 10.1021/acs.nanolett.0c02767

[22] R. Mo , H. Zhang , Y. Xu , X. Wu , S. Wang , Z. Dong , Y. Xia , D. Zheng , Q. Tan , Adv. Drug Delivery Rev 2023, 195, 114753. 10.1016/j.addr.2023.114753 36828300

[23] W. Liu , X. Zhai , X. Zhao , Y. Cai , X. Zhang , K. Xu , J. Weng , J. Li , X. Chen , Adv. Healthcare Mater. 2020, 12, 2300297. 10.1002/adhm.202300297 37114597

[24] J. M. Abdo , N. A. Sopko , S. M. Milner , Wound Med. 2020, 28, 100179.

[25] J. A. Bouwstra , R. W. J. Helder , A. El Ghalbzouri , Adv. Drug Delivery Rev. 2021, 175, 113802. 10.1016/j.addr.2021.05.012 34015420

[26] F. Rademacher , M. Simanski , R. Gläser , J. Harder , Exp. Dermatol. 2018, 27, 489. 29464787 10.1111/exd.13517

[27] M. H. Swaney , L. R. Kalan , A. R. Richardson , Infect. Immun. 2021, 89, 10.1128/iai.00695-20. PMC8090955 33468585

[28] H. Derakhshandeh , F. Aghabaglou , A. Mccarthy , A. Mostafavi , C. Wiseman , Z. Bonick , I. Ghanavati , S. Harris , C. Kreikemeier‐Bower , S. M. Moosavi Basri , J. Rosenbohm , R. Yang , P. Mostafalu , D. Orgill , A. Tamayol , Adv. Funct. Mater. 2020, 30, 1905544. 34354556 10.1002/adfm.201905544 PMC8336080

[29] M. R. Prausnitz , R. Langer , Nat. Biotechnol. 2008, 26, 1261. 18997767 10.1038/nbt.1504 PMC2700785

[30] E. Sparr , S. Björklund , Q. D. Pham , E. H. Mojumdar , B. Stenqvist , M. Gunnarsson , D. Topgaard , Curr. Opin. Colloid Interface Sci. 2023, 67, 101725.

[31] J. Wang , T. Wen , H. Chen , S. Huang , R. Guo , Y. Zheng , Z. Xiao , X. Shuai , Adv. Ther. 2024, 7, 2300362.

[32] I. Adigweme , M. Yisa , M. Ooko , E. Akpalu , A. Bruce , S. Donkor , L. B. Jarju , B. Danso , A. Mendy , D. Jeffries , A. Segonds‐Pichon , A. Njie , S. Crooke , E. El‐Badry , H. Johnstone , M. Royals , J. L. Goodson , M. R. Prausnitz , D. V. Mcallister , P. A. Rota , S. Henry , E. Clarke , Lancet 2024, 403, 1879. 38697170 10.1016/S0140-6736(24)00532-4 PMC11099471

[33] M. Farahani , A. Shafiee , Adv. Healthcare Mater. 2021, 10, 2100477. 10.1002/adhm.202100477 34174163

[34] R. F. Donnelly , T. R.R. Singh , A. D. Woolfson , Drug Delivery 2010, 17, 187. 20297904 10.3109/10717541003667798 PMC2906704

[35] X. Zhang , Z. Wang , H. Jiang , H. Zeng , N. An , B. Liu , L. Sun , Z. Fan , Sci. Adv. 2023, 9, eadh1415. 37450590 10.1126/sciadv.adh1415 PMC10348682

[36] D.D. Zhu , X. P. Zhang , B. L. Zhang , Y.Y. Hao , X. D. Guo , Adv. Ther. 2020, 3, 2000033.

[37] S. Wang , M. Zhao , Y. Yan , P. Li , W. Huang , Research 2023, 6, 0128. 37223469 10.34133/research.0128 PMC10202386

[38] S. Sharma , K. Hatware , P. Bhadane , S. Sindhikar , D. K. Mishra , Mater. Sci. Eng., C 2019, 103, 109717. 10.1016/j.msec.2019.05.002 31349403

[39] J. H. Jung , S. G. Jin , J. Pharm. Invest. 2021, 51, 503. 10.1007/s40005-021-00512-4 PMC7931162 33686358

[40] Z. Le , J. Yu , Y. J. Quek , B. Bai , X. Li , Y. Shou , B. Myint , C. Xu , A. Tay , Mater. Today 2023, 63, 137.

[41] T. Bauleth‐Ramos , N. El‐Sayed , F. Fontana , M. Lobita , M.‐A. Shahbazi , H. A. Santos , Mater. Today 2023, 63, 239.

[42] B. Chen , J. Wei , F. E. H. Tay , Y. T. Wong , C. Iliescu , Microsyst. Technol. 2008, 14, 1015.

[43] N. Tariq , M. W. Ashraf , S. Tayyaba , J. Pharm. Innovation 2022, 17, 1464.

[44] G. Anbazhagan , S. B. Suseela , R. Sankararajan , Drug Delivery Transl. Res. 2023, 13, 1813. 10.1007/s13346-023-01296-w 36807879

[45] A. Omolu , M. Bailly , R. M. Day , Drug Delivery 2017, 24, 942. 28618841 10.1080/10717544.2017.1337826 PMC8241162

[46] P. Makvandi , M. Kirkby , A. R. J. Hutton , M. Shabani , C. K. Y. Yiu , Z. Baghbantaraghdari , R. Jamaledin , M. Carlotti , B. Mazzolai , V. Mattoli , R. F. Donnelly , Nano‐Micro Lett. 2021, 13, 93. 10.1007/s40820-021-00611-9 PMC8006208 34138349

[47] I. Eş , A. Kafadenk , M. B. Gormus , F. Inci , Small 2023, 19, 2206510. 10.1002/smll.202206510 36929149

[48] C.‐W. Dong , J.‐Y. Jeon , H.‐M. Kang , W.‐T. Park , J. Mech. Sci. Technol. 2023, 37, 261.

[49] T.‐T. Liu , K. Chen , M. Pan , Med. Eng. Phys. 2017, 49, 148. 28888788 10.1016/j.medengphy.2017.08.012

[50] Á Cárcamo‐Martínez , B. Mallon , J. Domínguez‐Robles , L. K. Vora , Q. K. Anjani , R. F. Donnelly , Int. J. Pharm. 2021, 599, 120455. 33676993 10.1016/j.ijpharm.2021.120455

[51] K. B. Vinayakumar , P. G. Kulkarni , M.M. Nayak , N. S. Dinesh , G. M. Hegde , S. G. Ramachandra , K. Rajanna , J. Micromech. Microeng. 2016, 26, 065013.

[52] Y. Li , H. Zhang , R. Yang , Y. Laffitte , U. Schmill , W. Hu , M. Kaddoura , E. J. M. Blondeel , B. Cui , Microsyst. Nanoeng. 2019, 5, 41. 31636931 10.1038/s41378-019-0077-y PMC6799813

[53] S. N. Economidou , M. J. Uddin , M. J. Marques , D. Douroumis , W. T. Sow , H. Li , A. Reid , J. F. C. Windmill , A. Podoleanu , Addit. Manuf. 2021, 38, 101815.

[54] N. Tabassum , M. Alba , L. Yan , N. H. Voelcker , Adv. Ther. 2023, 6, 2200156.

[55] T. Liu , Y. Sun , W. Zhang , R. Wang , X. Lv , L. Nie , A. Shavandi , K. E. Yunusov , G. Jiang , J. Chem. Eng. 2024, 481, 148670.

[56] M. Parrilla , U. Detamornrat , J. Domínguez‐Robles , R. F. Donnelly , K. De Wael , Talanta 2022, 249, 123695. 35728453 10.1016/j.talanta.2022.123695

[57] Y. Dai , J. Nolan , E. Madsen , M. Fratus , J. Lee , J. Zhang , J. Lim , S. Hong , M.A. Alam , J. C. Linnes , H. Lee , C. H. Lee , ACS Appl. Mater. Interfaces 2023, 15, 56760. 10.1021/acsami.3c12740 38041570

[58] M. Parrilla , U. Detamornrat , J. Domínguez‐Robles , S. Tunca , R. F. Donnelly , K. De Wael , ACS Sens. 2023, 8, 4161. 37856156 10.1021/acssensors.3c01381

[59] M. G. Mcgrath , A. Vrdoljak , C. O’Mahony , J. C. Oliveira , A. C. Moore , A. M. Crean , Int. J. Pharm. 2011, 415, 140. 21664444 10.1016/j.ijpharm.2011.05.064

[60] T. N. Tarbox , A. B. Watts , Z. Cui , R. O. Williams , Drug Delivery Transl. Res. 2018, 8, 1828. 10.1007/s13346-017-0466-4 29288358

[61] A. Ullah , H. Khan , H. J. Choi , G. M. Kim , Polymers 2019, 11, 1834.31703443

[62] Y. Gao , M. Hou , R. Yang , L. Zhang , Z. Xu , Y. Kang , P. Xue , J. Mater. Chem. B 2019, 7, 7515. 31714572 10.1039/c9tb01994d

[63] Y. Chen , B. Z. Chen , Q. L. Wang , X. Jin , X. D. Guo , J. Controlled Release 2017, 265, 14. 10.1016/j.jconrel.2017.03.383 28344014

[64] Z. Sartawi , C. Blackshields , W. Faisal , J. Controlled Release 2022, 348, 186. 10.1016/j.jconrel.2022.05.045 35662577

[65] H. Chen , B. Wu , M. Zhang , P. Yang , B. Yang , W. Qin , Q. Wang , X. Wen , M. Chen , G. Quan , X. Pan , C. Wu , Drug Delivery Transl. Res. 2019, 9, 240. 10.1007/s13346-018-00593-z 30341765

[66] M. Yin , Y. Zeng , H.‐Q. Liu , W. Zhang , C. Wang , C. Chen , W. Li , ACS Appl. Mater. Interfaces 2023, 15, 17532. 36975753 10.1021/acsami.2c22814

[67] J. W. Lee , S‐O. Choi , E. I. Felner , M. R. Prausnitz , Small 2011, 7, 531. 21360810 10.1002/smll.201001091 PMC4143249

[68] A. J. Courtenay , E. Mcalister , M. T. C. Mccrudden , L. Vora , L. Steiner , G. Levin , E. Levy‐Nissenbaum , N. Shterman , M.‐C. Kearney , H. O. Mccarthy , R. F. Donnelly , J. Controlled Release 2020, 322, 177. 10.1016/j.jconrel.2020.03.026 PMC7262583 32200001

[69] M. Ali , S. Namjoshi , H. A. E. Benson , Y. Mohammed , T. Kumeria , J. Controlled Release 2022, 347, 561. 10.1016/j.jconrel.2022.04.043 35525331

[70] A. D. Permana , M. Mir , E. Utomo , R. F. Donnelly , Int. J. Pharm.: X 2020, 2, 100047. 32322819 10.1016/j.ijpx.2020.100047 PMC7168771

[71] K. Ita , Biomed. Pharmacother. 2017, 93, 1116. 28738520 10.1016/j.biopha.2017.07.019

[72] H. S. Min , Y. Kim , J. Nam , H. Ahn , M. Kim , G. Kang , M. Jang , H. Yang , H. Jung , Biomater. Adv. 2023, 145, 213248. 36610239 10.1016/j.bioadv.2022.213248

[73] A. Hou , G. Quan , B. Yang , C. Lu , M. Chen , D. Yang , L. Wang , H. Liu , X. Pan , C. Wu , Adv. Healthcare Mater. 2019, 8, 1900898. 10.1002/adhm.201900898 31583838

[74] L‐ Long , W. Liu , L. Li , C. Hu , S. He , L. Lu , J. Wang , L. Yang , Y‐ Wang , Nanoscale 2022, 14, 1285. 35006234 10.1039/d1nr07708b

[75] Y. Su , S. M. Andrabi , S. M. S. Shahriar , S. L. Wong , G. Wang , J. Xie , J. Controlled Release 2023, 356, 131. 10.1016/j.jconrel.2023.02.030 PMC10073311 36858263

[76] Y. Su , V. L. Mainardi , H. Wang , A. Mccarthy , Y. S. Zhang , S. Chen , J. V. John , S. L. Wong , R.R. Hollins , G. Wang , J. Xie , ACS Nano 2020, 14, 11775. 32840361 10.1021/acsnano.0c04527 PMC7673654

[77] Y. Liang , J. He , B. Guo , ACS Nano 2021, 15, 12687. 34374515 10.1021/acsnano.1c04206

[78] Z. Kopecki , N. E. Stevens , G. N. Yang , E. Melville , A. J. Cowin , Int. J. Mol. Sci. 2018, 19, 2014. 29996558 10.3390/ijms19072014 PMC6073877

[79] R. G. Frykberg , J. Banks , Adv. Wound Care 2015, 4, 560. 10.1089/wound.2015.0635 PMC4528992 26339534

[80] Z. Xu , S. Han , Z. Gu , J. Wu , Adv. Healthcare Mater. 2020, 9, 1901502. 10.1002/adhm.201901502 31977162

[81] M. Malone , G. Schultz , Br. J. Dermatol. 2022, 187, 159. 35587707 10.1111/bjd.21612

[82] H. Haidari , R. Bright , S. Garg , K. Vasilev , A. J. Cowin , Z. Kopecki , Biomedicines 2021, 9, 1182. 34572368 10.3390/biomedicines9091182 PMC8470956

[83] S. Wei , P. Xu , Z. Yao , X. Cui , X. Lei , L. Li , Y. Dong , W. Zhu , R. Guo , B. Cheng , Acta Biomater. 2021, 124, 205. 33524559 10.1016/j.actbio.2021.01.046

[84] E. P. Virgo , H. Haidari , Z. L. Shaw , L. Z. Y. Huang , T. L. Kennewell , L. Smith , T. Ahmed , S. J. Bryant , G. S. Howarth , S. Walia , A. J. Cowin , A. Elbourne , Z. Kopecki , Adv. Ther. 2023, 2300235.

[85] M. Lu , S. Li , X. Xiong , Z. Huang , B. Xu , Y. Liu , Q. Wu , N. Wu , H. Liu , D. Zhou , Adv. Funct. Mater. 2022, 32, 2208061.

[86] L. Flurin , Y. S. Raval , A. Mohamed , K. E. Greenwood‐Quaintance , E. J. Cano , H. Beyenal , R. Patel , Antimicrob. Agents Chemother. 2021, 65, 10.1128/aac.02007-20. PMC8092879 33649112

[87] P. Singh , S. M. Andrabi , U. Tariq , S. Gupta , S. Shaikh , A. Kumar , J. Chem. Eng. 2023, 457, 141359.

[88] M. Mirhaj , S. Labbaf , M. Tavakoli , A. Seifalian , Macromol. Biosci. 2022, 22, 2200014. 10.1002/mabi.202200014 35421269

[89] S. Li , S. Dong , W. Xu , S. Tu , L. Yan , C. Zhao , J. Ding , X. Chen , Adv. Sci. 2018, 5, 1700527. 10.1002/advs.201700527 PMC5980143 29876202

[90] J. Ouyang , Q. Bu , N. Tao , M. Chen , H. Liu , J. Zhou , J. Liu , B. Deng , N. Kong , X. Zhang , T. Chen , Y. Cao , W. Tao , Bioact. Mater. 2022, 18, 446. 35415296 10.1016/j.bioactmat.2022.03.033 PMC8971583

[91] H. Haidari , R. Bright , X. L. Strudwick , S. Garg , K. Vasilev , A. J. Cowin , Z. Kopecki , Acta Biomater. 2021, 128, 420. 33857695 10.1016/j.actbio.2021.04.007

[92] T. Peng , Y. Chen , W. Hu , Y. Huang , M. Zhang , C. Lu , X. Pan , C. Wu , Engineering 2023, 30, 170.

[93] K. J. Lee , Y. Xue , J. Lee , H‐J. Kim , Y. Liu , P. Tebon , E. Sarikhani , W. Sun , S. Zhang , R. Haghniaz , B. Çelebi‐Saltik , X. Zhou , S. Ostrovidov , S. Ahadian , N. Ashammakhi , M. R. Dokmeci , A. Khademhosseini , Adv. Funct. Mater. 2020, 30, 2000086. 33071712 10.1002/adfm.202000086 PMC7567343

[94] H.‐C. Flemming , J. Wingender , U. Szewzyk , P. Steinberg , S. A. Rice , S. Kjelleberg , Nat. Rev. Microbiol. 2016, 14, 563. 27510863 10.1038/nrmicro.2016.94

[95] Y.‐K. Wu , N.‐C. Cheng , C.‐M. Cheng , Trends Biotechnol. 2019, 37, 505. 30497871 10.1016/j.tibtech.2018.10.011

[96] Y. Su , J. T. Yrastorza , M. Matis , J. Cusick , S. Zhao , G. Wang , J. Xie , Adv. Sci. 2022, 9, 2203291. 10.1002/advs.202203291 PMC9561771 36031384

[97] H.‐C. Flemming , J. Wingender , Nat. Rev. Microbiol 2010, 8, 623. 20676145 10.1038/nrmicro2415

[98] S. Darvishi , S. Tavakoli , M. Kharaziha , H.H. Girault , C. F. Kaminski , I. Mela , Angew. Chem., Int. Ed. Engl. 2022, 61, e202112218. 34806284 10.1002/anie.202112218 PMC9303468

[99] G. Wang , W. Wang , Z. Chen , T. Hu , L. Tu , X. Wang , W. Hu , S. Li , Z. Wang , J. Chem. Eng. 2024, 482, 148938.

[100] Y. Su , S.S. M Shahriar , S. M. Andrabi , C. Wang , N. S. Sharma , Y. Xiao , S. L. Wong , G. Wang , J. Xie , Macromol. Biosci. 2024, 24, 2300519. 10.1002/mabi.202300519 38217528

[101] X. Zhang , G. Chen , Y. Liu , L. Sun , L. Sun , Y. Zhao , ACS Nano 2020, 14, 5901. 32315159 10.1021/acsnano.0c01059

[102] M. Sun , X. Zhong , M. Dai , X. Feng , C. Tang , L. Cao , L. Liu , Mater. Today Bio 2024, 24, 100945. 10.1016/j.mtbio.2024.100945 PMC10789642 38229885

[103] X. Yu , J. Zhao , D. Fan , J. Chem. Eng. 2022, 437, 135475.

[104] L. Chen , D. Fang , J. Zhang , X. Xiao , N. Li , Y. Li , M. Wan , C. Mao , J. Colloid Interface Sci. 2023, 647, 142. 37247478 10.1016/j.jcis.2023.05.080

[105] I. Woodhouse , S. Nejati , V. Selvamani , H. Jiang , S. Chittiboyina , J. Grant , Z. Mutlu , J. Waimin , N. S. Abutaleb , M. N. Seleem , R. Rahimi , ACS Appl. Bio Mater. 2021, 4, 5405. 10.1021/acsabm.1c00087 35006756

[106] P. S. Yarmolenko , E. J. Moon , C. Landon , A. Manzoor , D. W. Hochman , B. L. Viglianti , M. W. Dewhirst , Int. J. Hyperthermia 2011, 27, 320. 21591897 10.3109/02656736.2010.534527 PMC3609720

[107] D. He , X. Liu , J. Jia , B. Peng , N. Xu , Q. Zhang , S. Wang , L. Li , M. Liu , Y. Huang , X. Zhang , Y. Yu , G. Luo , Adv. Funct. Mater. 2024, 34, 2306357.

[108] L. E. González García , M. N. Macgregor , R. M. Visalakshan , N. Ninan , A.A. Cavallaro , A. D. Trinidad , Y. Zhao , A. J. D. Hayball , K. Vasilev , Chem. Commun. 2019, 55, 171. 10.1039/c8cc06035e 30418438

[109] X. Yang , M. Jia , Z. Li , Z. Ma , J. Lv , D. Jia , D. He , R. Zeng , G. Luo , Y. Yu , Int. J. Biol. Macromol. 2022, 215, 550. 35752336 10.1016/j.ijbiomac.2022.06.131

[110] Y. Su , A. McCarthy , S. L. Wong , R.R. Hollins , G. Wang , J. Xie , Adv. Healthcare Mater. 2021, 10, 2100135. 10.1002/adhm.202100135 PMC8222186 33887126

[111] Y. Gao , W. Zhang , Y. F. Cheng , Y. Cao , Z. Xu , L. Q. Xu , Y. Kang , P. Xue , Biomater. Sci. 2021, 9, 2244. 33514957 10.1039/d0bm02136a

[112] H. Haidari , Z. Kopecki , Microbiol. Aust. 2023, 44, 104.

[113] Y. Li , J.‐Y. Gong , P. Wang , H. Fu , F. Yousef , R. Xie , W. Wang , Z. Liu , D.‐W. Pan , X.‐J. Ju , L.‐Y. Chu , J. Colloid Interface Sci. 2024, 661, 123. 38295695 10.1016/j.jcis.2024.01.147

[114] H. Haidari , Z. Kopecki , R. Bright , A. J. Cowin , S. Garg , N. Goswami , K. Vasilev , ACS Appl. Mater. Interfaces 2020, 12, 41011. 32840353 10.1021/acsami.0c09414

[115] H. He , R. Cai , Y. Wang , G. Tao , P. Guo , H. Zuo , L. Chen , X. Liu , P. Zhao , Q. Xia , Int. J. Biol. Macromol. 2017, 104, 457. 28619637 10.1016/j.ijbiomac.2017.06.009

[116] S. Taheri , A. Cavallaro , S. N. Christo , L. E. Smith , P. Majewski , M. Barton , J. D. Hayball , K. Vasilev , Biomaterials 2014, 35, 4601. 24630091 10.1016/j.biomaterials.2014.02.033

[117] M. Zhao , M. Zhou , P. Gao , X. Zheng , W. Yu , Z. Wang , J. Li , J. Zhang , J. Mater. Chem. B 2022, 10, 1393. 35132982 10.1039/d1tb01991k

[118] Z. Kopecki , N. Ruzehaji , C. Turner , H. Iwata , R. J. Ludwig , D. Zillikens , D. F. Murrell , A. J. Cowin , J. Invest. Dermatol. 2013, 133, 1008. 23223144 10.1038/jid.2012.457

[119] H. An , Z. Gu , Z. Huang , T. Huo , Y. Xu , Y. Dong , Y. Wen , Colloids Surf., B 2024, 233, 113636. 10.1016/j.colsurfb.2023.113636 37979482

[120] Y. Zhang , S. Wang , Y. Yang , S. Zhao , J. You , J. Wang , J. Cai , H. Wang , J. Wang , W. Zhang , J. Yu , C. Han , Y. Zhang , Z. Gu , Nat. Commun. 2023, 14, 3431. 37301874 10.1038/s41467-023-39129-6 PMC10257705

[121] C. Zhao , Y. Li , J. Zhao , H. Li , J. Xu , Z. Gao , C. Ding , Y.‐Y. Song , ACS Nano 2023, 17, 13296. 37399243 10.1021/acsnano.3c01158

[122] R. Dong , B. Guo , Nano Today 2021, 41, 101290.

[123] H. Haidari , K. Vasilev , A. J. Cowin , Z. Kopecki , ACS Appl. Mater. Interfaces 2022, 14, 51744. 36356210 10.1021/acsami.2c15659

[124] R. F. Donnelly , M. J. Garland , D. I. J. Morrow , K. Migalska , T. R.R. Singh , R. Majithiya , A. D. Woolfson , J. Controlled Release 2010, 147, 333. 10.1016/j.jconrel.2010.08.008 20727929

[125] B. H. J. Gowda , M. G. Ahmed , A. Sahebkar , Y. Riadi , R. Shukla , P. Kesharwani , Biomacromolecules 2022, 23, 1519. 35274937 10.1021/acs.biomac.1c01691

[126] J. Yang , H. Zhang , T. Hu , C. Xu , L. Jiang , Y. Shrike Zhang , M. Xie , J. Chem. Eng. 2021, 426, 130561.

[127] L. Yang , Y. Gao , Q. Liu , W. Li , Z. Li , D. Zhang , R. Xie , Y. Zheng , H. Chen , X. Zeng , Small 2023, 20, 2307104. 10.1002/smll.202307104 37939306

[128] D. Lou , Q. Pang , X. Pei , S. Dong , S. Li , W‐ Tan , L. Ma , Biosens. Bioelectron. 2020, 162, 112275. 32392156 10.1016/j.bios.2020.112275

[129] A. Chanmugam , D. Langemo , K. Thomason , J. Haan , E.A. Altenburger , A. Tippett , L. Henderson , T. A. Zortman , Adv. Skin Wound Care 2017, 30, 406. 28817451 10.1097/01.ASW.0000522161.13573.62

[130] W. Zhu , J. Mei , X. Zhang , J. Zhou , D. Xu , Z. Su , S. Fang , J. Wang , X. Zhang , C. Zhu , Adv. Mater. 2022, 34, 2207961. 10.1002/adma.202207961 36239263

[131] M. Guo , Y. Wang , B. Gao , B. He , ACS Nano 2021, 15, 15316. 34533924 10.1021/acsnano.1c06279

[132] L. Fan , X. Zhang , M. Nie , Y. Xu , Y. Wang , L. Shang , Y. Zhao , Y. Zhao , Adv. Funct. Mater. 2022, 32, 2110746.

[133] J. Y. Li , Y. H. Feng , Y. T. He , L. F. Hu , L. Liang , Z. Q. Zhao , B. Z. Chen , X. D. Guo , Acta Biomater. 2022, 153, 308. 36055607 10.1016/j.actbio.2022.08.061

[134] S. Jiang , J. Bian , X. Shi , Y. Hu , Macromol. Biosci. 2023, 23, 2300018. 10.1002/mabi.202300018 37114319

[135] L. R. Bennison , C. N. Miller , R. J. Summers , A. M. B. Minnis , G. W. M. Sussman , Wound Pract. Res. 2017, 25, 63.

[136] S. Ono , R. Imai , Y. Ida , D. Shibata , T. Komiya , H. Matsumura , Burns 2015, 41, 820. 25468471 10.1016/j.burns.2014.10.023

[137] B. Mirani , Z. Hadisi , E. Pagan , S. M. H. Dabiri , A. van Rijt , L. Almutairi , I. Noshadi , D. G. Armstrong , M. Akbari , Adv. Healthcare Mater. 2023, 12, 2203233. 10.1002/adhm.202203233 PMC11468884 36929644

[138] H. Haidari , Z. Kopecki , A. T. Sutton , S. Garg , A. J. Cowin , K. Vasilev , Antibiotics 2021, 10, 49. 33466534 10.3390/antibiotics10010049 PMC7824857

[139] Y. Ma , H. Xu , B. Sun , S. Du , S. Cui , L. Zhang , N. Ding , D. Yang , ACS Appl. Mater. Interfaces 2021, 13, 59720. 34889592 10.1021/acsami.1c19681

[140] D. C. Singleton , A. Macann , W. R. Wilson , Nat. Rev. Clin. Oncol. 2021, 18, 751. 34326502 10.1038/s41571-021-00539-4

[141] Y. Zhang , T. Yue , W. Gu , A. Liu , M. Cheng , H. Zheng , D. Bao , F. Li , J.‐G. Piao , J. Nanobiotechnol. 2022, 20, 55. 10.1186/s12951-022-01262-7 PMC8800305 35093073

[142] X.‐L. Lei , K. Cheng , Y. Li , Z.‐T. Zhong , X.‐L. Hou , L.‐B. Song , F. Zhang , J.‐H. Wang , Y.‐D. Zhao , Q.‐R. Xu , J. Chem. Eng. 2023, 462, 142222.

[143] W. Li , N. Yang , X. Tan , Z. Liu , Y. Huang , R. Yuan , L. Liu , L. Ge , Colloids Surf., B 2023, 231, 113569. 10.1016/j.colsurfb.2023.113569 37826964

[144] A. Ullah , M. Jang , H. Khan , H. J. Choi , S. An , D. Kim , Y.‐R. Kim , U.‐K. Kim , G. M. Kim , Sens. Actuators, B 2021, 345, 130441.

[145] S. Zafar , M. S. Arshad , S. J. Rana , M. Patel , B. Yousef , Z. Ahmad , Int. J. Pharm. 2023, 640, 123003. 37146953 10.1016/j.ijpharm.2023.123003

[146] H. Haidari , L. Melguizo‐Rodríguez , A. J. Cowin , Z. Kopecki , Am. J. Physiol. Cell Physiol. 2023, 324, C29. 36409176 10.1152/ajpcell.00080.2022

[147] B. Wang , D. Zhao , Y. Li , X. Zhou , Z. Hui , X. Lei , L. Qiu , Y. Bai , C. Wang , J. Xia , Y. Xuan , P. Jiang , J. Wang , ACS Appl. Nano Mater. 2023, 6, 6891.

[148] P. W. Franks , M. I. Mccarthy , Science 2016, 354, 69. 27846494 10.1126/science.aaf5094

[149] Y. Zhu , J. Zhang , J. Song , J. Yang , Z. Du , W. Zhao , H. Guo , C. Wen , Q. Li , X. Sui , L. Zhang , Adv. Funct. Mater. 2020, 30, 1905493.

[150] F. Picard , B. Hersant , R. Bosc , J‐P. Meningaud , Wound Repair Regener. 2015, 23, 638. 10.1111/wrr.12317 26019054

[151] L. Prompers , M. Huijberts , J. Apelqvist , E. Jude , A. Piaggesi , K. Bakker , M. Edmonds , P. Holstein , A. Jirkovska , D. Mauricio , G. Ragnarson Tennvall , H. Reike , M. Spraul , L. Uccioli , V. Urbancic , K. Van Acker , J. Van Baal , F. Van Merode , N. Schaper , Diabetologia 2007, 50, 18. 17093942 10.1007/s00125-006-0491-1

[152] Y. Deng , Y. Gao , T. Li , S. Xiao , M. Adeli , R. D. Rodriguez , W. Geng , Q. Chen , C. Cheng , C. Zhao , ACS Nano 2023, 17, 2943. 36688804 10.1021/acsnano.2c11448

[153] J. Yu , Y. Zhang , Y. Ye , R. Disanto , W. Sun , D. Ranson , F. S. Ligler , J. B. Buse , Z. Gu , Proc. Natl. Acad. Sci. U.S.A. 2015, 112, 8260. 26100900 10.1073/pnas.1505405112 PMC4500284

[154] Z. Guo , H. Liu , Z. Shi , L. Lin , Y. Li , M. Wang , G. Pan , Y. Lei , L. Xue , J. Mater. Chem. B 2022, 10, 3501. 35416225 10.1039/d2tb00126h

[155] N. Syafika , S. B.A. Azis , C. K. Enggi , H. A. Qonita , T. R. A. Mahmud , A. Abizart , R. M. Asri , A. D. Permana , Mol. Pharmaceutics 2023, 20, 1269. 10.1021/acs.molpharmaceut.2c00936 36661193

[156] Q. Chen , Z. Xiao , C. Wang , G. Chen , Y. Zhang , X. Zhang , X. Han , J. Wang , X. Ye , M. R. Prausnitz , S. Li , Z. Gu , ACS Nano 2022, 16, 18223. 36322923 10.1021/acsnano.2c05687 PMC10738036

[157] X.‐X. Yang , Y.‐L. Chen , P.‐F. Feng , C.‐C. Wang , X.‐K. Li , L.‐L. Liu , Y. Tang , Mater. Chem. Front. 2022, 6, 680.

[158] M. Lu , X. Zhang , D. Xu , N. Li , Y. Zhao , Adv. Mater. 2023, 35, 2211330. 10.1002/adma.202211330 36905684

[159] Y. Wang , H. Liu , X. Yang , Z. Shi , J. Li , L. Xue , S. Liu , Y. Lei , Smart Mater. Med. 2023, 4, 69.

[160] J. Chen , Y. Liu , G. Cheng , J. Guo , S. Du , J. Qiu , C. Wang , C. Li , X. Yang , T. Chen , Z. Chen , Small 2022, 18, 2201300. 10.1002/smll.202201300 35678523

[161] G. Saravanakumar , J. Kim , W. J. Kim , Adv. Sci. 2017, 4, 1600124. 10.1002/advs.201600124 PMC5238745 28105390

[162] P. L. Thi , Y. Lee , D. L. Tran , T.T. H Thi , J. I. Kang , K. M. Park , K. D. Park , Acta Biomater. 2020, 103, 142. 31846801 10.1016/j.actbio.2019.12.009

[163] Y. Wu , Y. Wang , L. Long , C. Hu , Q. Kong , Y. Wang , J. Controlled Release 2022, 341, 147. 10.1016/j.jconrel.2021.11.027 34813880

[164] C. Geng , S. He , S. Yu , H. M. Johnson , H. Shi , Y. Chen , Y. K. Chan , W. He , M. Qin , X. Li , Y. Deng , Adv. Mater. 2024, 2310599. 10.1002/adma.202310599 38300795

[165] D. Bi , F. Qu , W. Xiao , J. Wu , P. Liu , H. Du , Y. Xie , H. Liu , L. Zhang , J. Tao , Y. Liu , J. Zhu , ACS Nano 2023, 17, 4346. 36847798 10.1021/acsnano.2c08979

[166] J. Yang , Z. Chu , Y. Jiang , W. Zheng , J. Sun , L. Xu , Y. Ma , W. Wang , M. Shao , H. Qian , Adv. Healthcare Mater. 2023, 12, 2300725. 10.1002/adhm.202300725 37086396

[167] X. Yu , J. Zhao , X. Ma , D. Fan , J. Chem. Eng. 2023, 465, 142933.

[168] Y. Zhang , P. Feng , J. Yu , J. Yang , J. Zhao , J. Wang , Q. Shen , Z. Gu , Adv. Ther. 2018, 1, 1800035.

[169] H. Ding , Y. Cui , J. Yang , Y. Li , H. Zhang , S. Ju , X. Ren , C. Ding , J. Zhao , J. Controlled Release 2023, 360, 365. 10.1016/j.jconrel.2023.03.060 37331606

[170] S. Lyu , Z. Dong , X. Xu , H.‐P. Bei , H.‐Y. Yuen , C.‐W. J. Cheung , M.‐S. Wong , Y. He , X. Zhao , Bioact. Mater. 2023, 27, 303. 37122902 10.1016/j.bioactmat.2023.04.003 PMC10140753

[171] R. Haghniaz , H.‐J. Kim , H. Montazerian , A. Baidya , M. Tavafoghi , Y. Chen , Y. Zhu , S. Karamikamkar , A. Sheikhi , A. Khademhosseini , Bioact. Mater. 2023, 23, 314. 36439081 10.1016/j.bioactmat.2022.08.017 PMC9692134

[172] Z. Chen , X. Hu , Z. Lin , H. Mao , Z. Qiu , K. Xiang , T. Ke , L. Li , L. Lu , L. Xiao , ACS Appl. Mater. Interfaces 2023, 15, 43309. 37688542 10.1021/acsami.3c06800

[173] S. Gao , Y. Rao , X. Wang , Q. Zhang , Z. Zhang , Y. Wang , J. Guo , F. Yan , Adv. Mater. 2024, 36, 2307585. 10.1002/adma.202307585 38307004

[174] J. Xu , S. Lin , H. Chen , G. Yang , M. Zhou , Y. Liu , A. Li , S. Yin , X. Jiang , Adv. Healthcare Mater. 2024, 13, 2304315. 10.1002/adhm.202304315 38261729

[175] S. Zhang , T. Jiang , F. Han , L. Cao , M. Li , Z. Ge , H. Sun , H. Wu , W. Wu , N. Zhou , M. L. Akhtar , H. Jiang , J. Chem. Eng. 2024, 480, 148347.

[176] Y. Hu , E. Chatzilakou , Z. Pan , G. Traverso , A. K. Yetisen , Adv. Sci. 2024, 11, 2306560. 10.1002/advs.202306560 PMC10966570 38225744

[177] N. Tang , Y. Zheng , D. Cui , H. Haick , Adv. Healthcare Mater. 2021, 10, 2101292. 10.1002/adhm.202101292 34310078

[178] H. Zhang , X. Sun , J. Wang , Y. Zhang , M. Dong , T. Bu , L. Li , Y. Liu , L. Wang , Adv. Funct. Mater. 2021, 31, 2100093.

[179] J. Jiang , J. Ding , X. Wu , M. Zeng , Y. Tian , K. Wu , D. Wei , J. Sun , Z. Guo , H. Fan , J. Mater. Chem. B 2023, 11, 4934. 37194435 10.1039/d3tb00099k

[180] H. Cheng , Z. Shi , K. Yue , X. Huang , Y. Xu , C. Gao , Z. Yao , Y. S. Zhang , J. Wang , Acta Biomater. 2021, 124, 219. 33556605 10.1016/j.actbio.2021.02.002

[181] J. L. Ramirez‐Garcialuna , M. A. Martinez‐Jimenez , R. D. J. Fraser , R. Bartlett , A. Lorincz , Z. Liu , G. Saiko , G. K. Berry , Front. Med. 2023, 10, 1165281. 10.3389/fmed.2023.1165281 PMC10483069 37692790

[182] Y.‐E. Kang , K.‐Y. Seong , S.‐G. Yim , Y. Lee , S.‐M. An , S. C. Kim , K. Kim , B.‐S. An , K.‐S. Lee , S. Y. Yang , Biosens. Bioelectron. 2020, 163, 112281. 32568694 10.1016/j.bios.2020.112281

[183] J. Yang , X. Gong , S. Chen , Y. Zheng , L. Peng , B. Liu , Z. Chen , X. Xie , C. Yi , L. Jiang , ACS Sens. 2023, 8, 1241. 36821704 10.1021/acssensors.2c02635

[184] S. Latiyan , T. S. S. Kumar , M. Doble , J. F. Kennedy , Int. J. Biol. Macromol. 2023, 244, 125358. 37330091 10.1016/j.ijbiomac.2023.125358

[185] Z. Le , J. Yu , Y. J. Quek , B. Bai , X. Li , Y. Shou , B. Myint , C. Xu , A. Tay , Mater. Today 2023, 63, 137.

[186] M. T. C. Mccrudden , E. Mcalister , A. J. Courtenay , P. González‐Vázquez , T. R. Raj Singh , R. F. Donnelly , Exp. Dermatol. 2015, 24, 561. 25865925 10.1111/exd.12723

[187] Y. Hiraishi , T. Nakagawa , Y.‐S. Quan , F. Kamiyama , S. Hirobe , N. Okada , S. Nakagawa , Int. J. Pharm. 2013, 441, 570. 23137695 10.1016/j.ijpharm.2012.10.042

[188] R. Pireddu , M. Schlich , S. Marceddu , D. Valenti , E. Pini , A. M. Fadda , F. Lai , C. Sinico , Pharmaceutics 2020, 12, 1140. 33255623 10.3390/pharmaceutics12121140 PMC7760567

[189] R. S. J. Ingrole , E. Azizoglu , M. Dul , J. C. Birchall , H. S. Gill , M. R. Prausnitz , Biomaterials 2021, 267, 120491. 33217629 10.1016/j.biomaterials.2020.120491 PMC8042615

[190] N. Sultana , A. Waheed , A. Ali , S. Jahan , M. Aqil , Y. Sultana , M. Mujeeb , Expert Opin. Drug Delivery 2023, 20, 739. 10.1080/17425247.2023.2201494 37038271

[191] K. Castorino , S. Polsky , G. O'malley , C. Levister , K. Nelson , C. Farfan , S. Brackett , S. Puhr , C. J. Levy , Diabetes Technol. Ther. 2020. 22, 943. 32324061 10.1089/dia.2020.0085 PMC7757524

[192] Y. Liu , Q. Yu , X. Luo , L. Yang , Y. Cui , Microsyst. Nanoeng. 2021, 7, 75. 34631143 10.1038/s41378-021-00302-w PMC8481261

[193] A. Panda , V. A. Matadh , S. Suresh , H. N. Shivakumar , S. N. Murthy , Drug Delivery Transl. Res. 2022, 12, 67. 10.1007/s13346-021-00922-9 33629222

[194] M. R. Babu , S. Vishwas , R. Khursheed , V. Harish , A. B. Sravani , F. Khan , B. Alotaibi , A. Binshaya , J. Disouza , P. S. Kumbhar , V. Patravale , G. Gupta , R. Loebenberg , M. F. Arshad , A. Patel , S. Patel , K. Dua , S. K. Singh , Drug Deliv. Transl. Res. 2024, 14, 1393. 38036849 10.1007/s13346-023-01475-9

[195] E. Larrañeta , R. E. M. Lutton , A. D. Woolfson , R. F. Donnelly , Mater. Sci. Eng.: R: Rep. 2016, 104, 1.

[196] A. Sadeqi , G. Kiaee , W. Zeng , H. Rezaei Nejad , S. Sonkusale , Sci. Rep. 2022, 12, 1853. 35115643 10.1038/s41598-022-05912-6 PMC8813900

[197] R. F. Donnelly , T. R.R. Singh , M.M. Tunney , D. I. J. Morrow , P. A. Mccarron , C. O’Mahony , A. D. Woolfson , Pharm. Res. 2009, 26, 2513. 19756972 10.1007/s11095-009-9967-2 PMC2900181

[198] H. X. Nguyen , C. N. Nguyen , Pharmaceutics 2023, 15.

[199] K. Cheung , D. B. Das , Drug Delivery 2016, 23, 2338. 25533874 10.3109/10717544.2014.986309

[200] K. Ita , Pharmaceutics 2015, 7, 90. 26131647 10.3390/pharmaceutics7030090 PMC4588187

[201] M. J. Garland , K. Migalska , T. M. T. Mahmood , T. R.R. Singh , A. D. Woolfson , R. F. Donnelly , Expert Rev. Med. Devices 2011, 8, 459. 21728732 10.1586/erd.11.20

[202] T. Waghule , G. Singhvi , S. K. Dubey , M.M. Pandey , G. Gupta , M. Singh , K. Dua , Biomed. Pharmacother. 2019, 109, 1249. 30551375 10.1016/j.biopha.2018.10.078

[203] K. J. Lee , S.S. Jeong , D. H. Roh , D. Y. Kim , H.‐K. Choi , E. H. Lee , Int. J. Pharm. 2020, 573, 118778. 31678394 10.1016/j.ijpharm.2019.118778

[204] M. Azmana , S. Mahmood , A. R. Hilles , U. K. Mandal , K. A. Saeed Al‐Japairai , S. Raman , J. Drug Delivery Sci. Technol. 2020, 60, 101877.

[205] Y. Jin , Y. Lu , X. Jiang , M. Wang , Y. Yuan , Y. Zeng , L. Guo , W. Li , Bioact. Mater. 2024, 38, 292. 38745591 10.1016/j.bioactmat.2024.05.008 PMC11091528

[206] D. Yu , L. Chen , T. Yan , Y. Zhang , X. Sun , G. Lv , S. Zhang , Y. Xu , C. Li , Adv. Healthcare Mater. 2024, 2301985. 10.1002/adhm.202301985 38776526

